# RNA Methyltransferase METTL16’s Protein Domains Have Differential Functional Effects on Cell Processes

**DOI:** 10.3390/cimb45070346

**Published:** 2023-06-29

**Authors:** Emily S. Talic, Ashley Wooten, Tonya N. Zeczycki, Kyle D. Mansfield

**Affiliations:** 1 Biochemistry and Molecular Biology Department, Brody School of Medicine, East Carolina University, Greenville, NC 27834, USA; satterwhitee18@students.ecu.edu (E.S.T.); zeczyckit@ecu.edu (T.N.Z.); 2 Mass Spectrometry Core Facility, Brody School of Medicine, East Carolina University, Greenville, NC 27834, USA

**Keywords:** METTL16, N6-methyladenosine, m6A, methyltransferase, cell cycle, RNA-binding protein

## Abstract

METTL16, a human m6A RNA methyltransferase, is currently known for its modification of U6 and MAT2A RNAs. Several studies have identified additional RNAs to which METTL16 binds, however whether METTL16 modifies these RNAs is still in question. Moreover, a recent study determined that METTL16 contains more than one RNA-binding domain, leaving the importance of each individual RNA-binding domain unknown. Here we examined the effects of mutating the METTL16 protein in certain domains on overall cell processes. We chose to mutate the N-terminal RNA-binding domain, the methyltransferase domain, and the C-terminal RNA-binding domain. With these mutants, we identified changes in RNA-binding ability, protein and RNA expression, cell cycle phase occupancy, and proliferation. From the resulting changes in RNA and protein expression, we saw effects on cell cycle, metabolism, intracellular transport, and RNA processing pathways, which varied between the METTL16 mutant lines. We also saw significant effects on the G1 and S phase occupancy times and proliferative ability with some but not all the mutants. We have therefore concluded that while METTL16 may or may not m6A-modify all RNAs it binds, its binding (or lack of) has a significant outcome on a variety of cell processes.

## 1. Introduction

Modification of adenosine to methyl-6-adenosine (m6A) in RNA has been extensively studied in multiple species of prokaryotes, eukaryotes, and viruses for several decades [1,2,3,4]. This modification, which has been shown in mRNA, lncRNA, rRNA, miRNA, snRNA, snoRNA, and tRNA, has been proven to affect the half-life [5,6,7], translation efficiency [8,9,10,11,12,13,14], splicing [15,16,17], storage [18], or (in the case of miRNA) maturation of the RNA [19,20]. These effects are brought about either by interactions with m6A RNA-binding proteins or by fine-tuning the thermodynamics of the RNA structure [21,22,23]. Furthermore, m6A modification is one of the most common modifications in cellular RNA and currently considered the most common modification in mRNA [2].

The m6A modification is produced by m6A RNA methyltransferases, colloquially termed “m6A writers”. These m6A writers catalyze the transfer of a methyl group from a S-adenosylmethionine molecule to the target adenosine’s 6th base position [24]. There are more than 60 known RNA methyltransferases in humans, however most of these have been shown to modify tRNAs and rRNAs [25]. Currently, there are two established human m6A RNA methyltransferases shown to modify mRNA, which are the METTL3/14 complex and METTL16. METTL3/14 is the more studied of the two and is known to modify many mRNAs [5,19,26,27,28,29]. RNA substrates for this m6A writer complex need to be in a single-stranded conformation and contain a “DRACH” consensus sequence (D = A/U/G, R= A/G, H = A/U/C; underlined A is the modified adenosine) [2,30,31,32,33]. The few RNAs that have been observed in complex with METTL16 have all shown a double-stranded conformation and contain a “UACAGAGAA” consensus sequence (underlined A is the modified adenosine) [34,35]. RNAs modified by the METTL3/14 complex have a wide variety of functions, therefore it is unsurprising that dysregulation of this complex is frequently observed in multiple diseases including several cancers [8,36,37,38,39,40,41,42]. While METTL16 is the lesser studied, its dysregulation has been observed in several cancers as well [43,44,45,46,47,48,49,50,51,52].

Proteins known as “m6A readers” have a high affinity for binding RNAs that are m6A modified. After binding to m6A RNAs, these proteins are directly or indirectly responsible for the effects of m6A modification mentioned previously (such as splicing). Currently known m6A readers include YTHDF1/2/3 [7,12,53], YTHDC1/2 [54,55,56,57], HNRNPA2B1 [58,59], HNRNPC [23], HNRNPG [60], IGF2BP1/2/3 [18], and others. The m6A modification can be removed from the RNA by m6A demethylase also known as “m6A erasers”, which include ALKBH5 [61] and FTO [62,63]. These enzymes transfer the methyl group from the RNA to a 2-oxoglutarate molecule. The balance between adding and removing this modification from cell RNAs is a complex and delicate system that can become upset by dysregulation of any of the enzymes mentioned in this pathway.

As an m6A RNA methyltransferase, METTL16 has gained attention as a potential therapeutic for several diseases associated with cellular m6A dysregulation. It has been shown by several groups to methylate MAT2A mRNA and U6 snRNA, and to bind MALAT1 lncRNA [34,35,57,64,65,66,67]. Global effects on cellular m6A levels with knockdown or knock out of METTL16 have also been reported [35,44]. Moreover, widespread RNA expression changes have been seen in response to changes in METTL16 protein levels [35]. Despite a number of studies, there unfortunately seems to be little overlap between the m6A and RNA expression datasets. This could be due to different cell types being used, availability of SAM for modification to occur (which is produced by MAT2 enzyme), or a difference in the extent and/or duration of METTL16 knockdown. Furthermore, METTL16 has been implicated in a large number of cancer studies with both oncogenic and tumor-suppressive effects associated with changes in METTL16 expression levels [43,44,45,46,61,62,63,64,65,66,67,68,69,70,71,72,73]. For example, the database OncoMX demonstrates that METTL16 RNA has shown significant upregulation in kidney and colorectal cancers, but significant downregulation in uterine, bladder, prostate, breast, and lung cancers [74].

**Figure 1:**
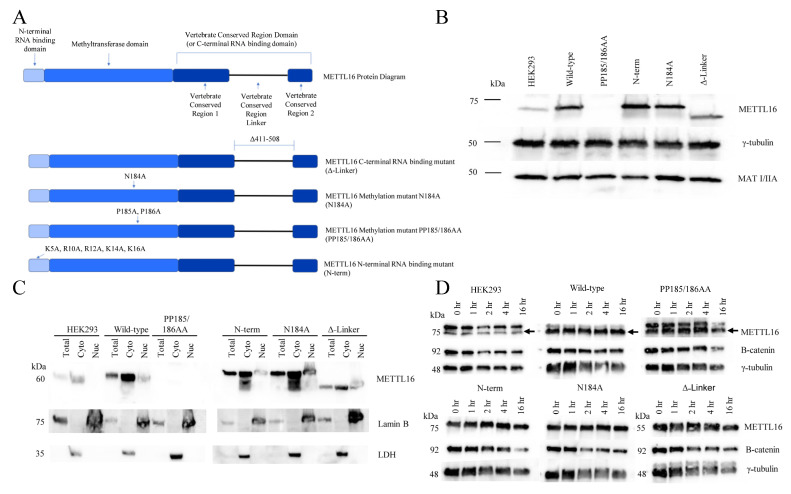
Human METTL16 mutants used in this study. (**A**) Protein diagrams of each METTL16 mutant produced for this study. Sizes of each domain are approximate to actual size. (**B**) Western blot of HEK293 transgenic METTL16 mutant cell lines with endogenous METTL16 removed via CRISPR-Cas9 using METTL16 antibody. Mutant PP185/186AA cell line shows no expression of METTL16. MAT2A protein levels are shown for reference and gamma—tubulin is shown as a loading control. (**C**) Cellular localization of METTL16 in HEK293 and HEK293 transgenic METTL16 mutant cell lines via Western blot after removal of endogenous METTL16 via CRISPR-Cas9. PP185/186AA METTL16 mutant CRISPR clonal line shows no detectable METTL16 protein. Lactate dehydrogenase (LDH) was used to confirm cytoplasmic content, Lamin B was used to confirm nuclear content. (**D**) Western blots of HEK293 and each HEK293 METTL16 mutant CRISPR clonal line used in this study subjected to 0, 1, 2, 4, or 16 h of cycloheximide to determine half-life of endogenous and transgenic mutant METTL16.

As shown in Figure 1A, METTL16 has a number of well-defined protein domains. The methyltransferase domain contains the conserved Rossmann-like fold of class I methyltransferases [75]. A hydrophobic pocket facilitates the proper orientation of SAM while the canonical “NPPF” amino acid sequence stabilizes the methionine [64]. The N-terminal RNA-binding domain of METTL16 forms a projection from the methyltransferase domain and acts as a “claw” to further stabilize the RNA in the methyltransferase RNA groove [75]. It contains a number of polar and positive-charged amino acids that form a groove to allow double-stranded RNA placement [75,76]. The C-terminus of METTL16 comprises the vertebrate conserved region (VCR) domain. The domain is speculated to function as a splicing director for MAT2A RNA [35], but it also plays a role in the methyltransferase reaction as well. Because of the presumed motion of this domain, it is believed to bind near the target adenosine (in a double-stranded region) to bend the RNA and allow better access of the adenosine to the core methyltransferase domain [66].

Because METTL16 studies have shown a wide variety of effects due to knockdown, knockout, mutation, or overexpression, and not all METTL16’s protein domains are fully characterized, we decided to further study the functional effects of individual domains of METTL16 to determine their specific duties. We chose to mutate the N-terminal RNA-binding domain, the methyltransferase domain, or the VCR RNA-binding domain using previously published mutations. We stably expressed the mutated METTL16 in HEK293 cells. The endogenous METTL16 was then removed from these same cells so that only the mutated version of METTL16 remained. These cells lines were established, confirmed, and analyzed for changes in the RNA-binding abilities of METTL16, global RNA expression, protein expression, and cell cycle effects.

## 2. Materials and Methods

### 2.1. Plasmid Production

METTL16-Myc-FLAG plasmid, METTL16 CRISPR-Cas9 plasmid, and scrambled CRISPR-Cas9 plasmid were purchased from Origene and Genecopoeia (Supplemental Table S3). Mutations in the METTL16 plasmid coding sequence were introduced using the Q5 Site-Directed Mutagenesis Kit (New England Biolabs, Ipswich, MA, USA) and primers found in Supplemental Table S2. Plasmids were amplified with transformation of FB5α *E. coli* cells (Thermo Fisher Scientific Inc., Waltham, MA, USA) via incubation for 30 s at 45 °C, then left for 5 min on ice. *E. coli* suspension was then adjusted to 1 mL with SOC Outgrowth Media (New England Biolabs) and incubated for 1 h at 37 °C on a shaker at 250 rpm. Bacterial cells were spun down, resuspended in 50 µL of SOC Outgrowth Media, and spread on a Luria Broth (LB) agar plate (Luria Broth from Fisher Scientific Inc., Hampton, NH, USA, Agar from Sigma-Aldrich, Burlington, MA, USA-Aldrich, Burlington, MA, USA) containing the appropriate antibiotic for selection. Plates were incubated at 37 °C overnight. Colonies were selected from plates after growth, suspended in 3 mL of LB media with appropriate antibiotic, and grown for approximately 16 h at 37 °C on a shaker at 250 rpm. Plasmids were harvested from bacteria using the GeneJET Plasmid Miniprep Kit (Thermo Fisher Scientific Inc., Waltham, MA, USA) according to manufacturer protocols. Some of the *E. coli* was kept at 4 °C for later use. The mini-prepped plasmids were sequenced with Sanger sequencing using primers directed at key sequences in the plasmids. Once verified, the stored *E. coli* cell suspensions were adjusted with another 3 mL of LB media and respective antibiotic, grown overnight at 37 °C, and then added to 250 mL of LB with antibiotic, which was again grown overnight at 37 °C. Plasmids from these cells were harvested using the NucleoBond Xtra Midi Kit (Macherey-Nagel, Düren, Nordrhein-Westfalen, Germany) according to manufacturer protocols. When appropriate, a final concentration of 100 µg/mL of ampicillin (Sigma-Aldrich, Burlington, MA, USA) or 100 µg/mL of kanamycin (Sigma-Aldrich, Burlington, MA, USA) was used for selection. Plasmid concentrations were determined with a Nanodrop 2000 spectrophotometer.

### 2.2. Tissue Culture

HEK293 cells were obtained directly from American Type Culture Collection (ATCC). Cells were cultured at 37 °C, 5% CO_2_, in Dulbecco’s Modification of Eagle’s Medium 4.5 g/L glucose (Corning, Manassas, VA, USA) supplemented with 10% fetal bovine serum (Gemini Bio, Sacramento, CA, USA), 2mM L-glutamine (Corning, Manassas, VA, USA), and 1X Penicillin/Streptomycin (Corning, Manassas, VA, USA). When appropriate, G418 (Sigma-Aldrich, Burlington, MA, USA) was used in culture at 0.5mg/mL for selection and 0.25 mg/mL for maintenance; puromycin (Sigma-Aldrich, Burlington, MA, USA) was used in culture at 7.5 µg/mL for selection and 3.75 µg/mL for maintenance.

### 2.3. Plasmid Transfection

For clone production, cells were plated at 0.5 × 10^6^/well in a 6-well plate (CytoOne, USA Scientific, Ocala, FL, USA) the day before transfection. On the day of transfection, 2 µg of plasmid was transfected with Opti-MEM (Gibco, Thermo Fisher Scientific Inc., Waltham, MA, USA) and Lipofectamine 2000 (Invitrogen, Thermo Fisher Scientific Inc., Waltham, MA, USA) according to manufacturer protocols. The plasmid mixture was incubated with cells overnight, and the media were changed the next day. Antibiotic selection was started 24 h after initial transfection time. Cell colonies were selected once visible after approximately 1 week, grown separately, and screened for proper plasmid expression and integration using Western blotting. Cells transfected for transient overexpression were plated in a 10-cm cell culture dish (CytoOne, USA Scientific, Ocala, FL, USA) with 2.5 × 10^6^ cells, transfected with 10 µg of plasmid, and harvested one to two days after transfection.

### 2.4. Western Blots

Cells were harvested using 0.05% Trypsin (Corning, Manassas, VA, USA), quenched with media, washed with 1X Dulbecco’s Phosphate Buffered Saline (DPBS; Corning, Manassas, VA, USA), and then lysed with a Whole-Cell Extract buffer (WCEB; 150 mM NaCl (Mallinckrodt, Webster Groves, MO, USA), 10 mM Tris pH = 7.6 (Corning, Manassas, VA, USA), 0.1% SDS (Sigma-Aldrich, Burlington, MA, USA), 5 mM EDTA (Ambion, Austin, TX, USA), 1 × protease inhibitors (Pierce, Thermo Fisher Scientific Inc., Waltham, MA, USA ), incubated on ice for about 5 min, and stored at −80 °C until use. After thawing on ice, extracts were sonicated, and protein was quantified with the BCA kit (Thermo Fisher Scientific Inc., Waltham, MA, USA) in triplicate according to manufacturer protocols as well as with the Promega GloMax Multi Detection plate reader. Unless otherwise stated, 35 µg of protein was loaded from each sample with 5 loading buffers (250 mM Tris-HCl pH = 6.8, 10% SDS *w*/*v*, 500 mM DTT, 750 µM Bromophenol Blue sodium salt (Sigma-Aldrich, Burlington, MA, USA), 50% Glycerol *v*/*v* (Sigma-Aldrich, Burlington, MA, USA), and 1% β-Mercaptoethanol (Sigma-Aldrich, Burlington, MA, USA) into the Western gel. For mini gels (Bio-Rad Laboratories, Hercules, CA, USA), the gel was electrophoresed at room temperature at 250 V for 25 min in 1 Tris-Glycine-SDS buffer (Bio-Rad Laboratories, Hercules, CA, USA). Protein was transferred to an Amersham Protran 0.2-µm nitrocellulose membrane (GE Healthcare, Chicago, IL, USA) at room temperature with an ice pack and stir bar at 300mA for 1 h in 1 Tris-Saline +20% methanol (Fisher Scientific Inc., Hampton, NH, USA) buffer. For large separation gels (20 cm), the gel was electrophoresed at 4 °C at approximately 150 V for several hours in 1 Tris-Glycine-SDS buffer (Bio-Rad Laboratories, Hercules, CA, USA) until proper separation was achieved. Protein was transferred to a nitrocellulose membrane at 4 °C at 300 mA for 1 h in 1 Tris-Saline +20% methanol buffer. After transfer, membranes were handled in the same manner. Membranes were blocked with 5% nonfat milk (LabScientific, Fisher Scientific Inc., Hampton, NH, USA) in 1 TBS-T (Tris-buffered saline with 0.1% Tween 20 (Sigma-Aldrich, Burlington, MA, USA)) at room temperature for 1 h on a shaker, and then incubated in appropriate primary antibody suspended in 5% nonfat milk or 5% bovine serum albumin (Fisher Scientific Inc., Hampton, NH, USA) in 1 TBS-T (with 0.01% NaN_3_ (Sigma-Aldrich, Burlington, MA, USA)) overnight at 4 °C on a shaker (See Supplemental Table S1 for antibody details). The next day, membranes were washed at room temperature on a shaker with 1 TBS-T 3 times with incubations for each wash at least 5 min. Membranes were then incubated with 5% nonfat milk in 1 TBS-T with appropriate 1:10,000 secondary HRP antibody (GE Healthcare, Chicago, IL, USA) at room temperature for 1 h on a shaker. Washes were repeated as described with 1 TBS-T. Western blot was imaged with the Clarity Western ECL Substrate kit (Bio-Rad Laboratories, Hercules, CA, USA) according to manufacturer protocols using MyECL Imager (Thermo Fisher Scientific Inc., Waltham, MA, USA) or iBright CL1500 Imager (Invitrogen, Thermo Fisher Scientific Inc., Waltham, MA, USA). Original, uncropped Western blots for each Figure can be found in Supplemental Figure S4.

### 2.5. Cycloheximide Treatment

Cells were lifted with 0.05% Trypsin (Corning, Manassas, VA, USA), counted with Trypan Blue (Corning, Manassas, VA, USA), and plated in a 6-well dish the day before treatment at 0.4 × 10^6^ cells/well. On the day of treatment, all wells were treated with a final concentration of 25 µg/mL of cycloheximide (Sigma-Aldrich, Burlington, MA, USA) and harvested at the time points specified in WCEB with 1 protease inhibitor. Protein concentration was quantified using the BCA kit from Thermo Fisher Scientific Inc. (Waltham, MA, USA), and Western blots were produced as listed in the Western blot section.

### 2.6. Cell Biochemical Fractionation

Cells were lifted with 0.05% Trypsin, quenched with media, centrifuged at 1500× *g* for 5 min, rinsed with 1 mL of DPBS, and centrifuged again after moving 100 µL to a separate tube. DPBS was removed. The 100 µL of cells was resuspended in 100 µL of a Total Lysis Buffer (TLB) (50 mM Tris pH = 8 (Sigma-Aldrich, Burlington, MA, USA), 150 mM NaCl, 0.1% SDS, 1% NP40 (Fluka Analytical, Charlotte, NC, USA), 0.5% sodium deoxycholate (Sigma-Aldrich, Burlington, MA, USA)) + protease inhibitors, and the 900 µL of cells was resuspended in 100 µL of a Hypotonic Lysis Buffer (HLB) (10 mM Tris pH = 8, 10 mM NaCl, 1.5 mM MgCl_2_ (Sigma-Aldrich, Burlington, MA, USA)) + protease inhibitors. Both were frozen at −80 °C overnight to further the lysis. The HLB cells were then thawed on ice, vortexed for a few seconds to lyse, and centrifuged at 1500× *g* for 5 min. The resulting supernatant was the cytoplasmic fraction which was moved to a separate tube. The pellet was the nuclear fraction and was resuspended in 100 µL of TLB + protease inhibitors. Nuclear and total fractions were sonicated to break up DNA, and equal amounts were loaded for Western blotting after addition of a loading buffer. Western blots were produced as listed in the Western blot section.

### 2.7. RNA Extraction

Cells were collected and then lysed with 1 mL of TRIzol (Thermo Fisher Scientific Inc., Waltham, MA, USA). RNA was isolated according to manufacturer protocols and kept on ice thereafter. RNA concentrations were determined with a Qubit 4 Fluorometer (Thermo Fisher Scientific Inc., Waltham, MA, USA) and stored in RNase-free H_2_O at −80 °C until use.

### 2.8. Immunoprecipitation

A confluent 10-cm cell culture dish was harvested by gently rinsing the cells off with 1 mL of DPBS. Cells were spun down and resuspended in 125–250 µL of a Polysome Lysis Buffer (100 mM KCl (Sigma-Aldrich, Burlington, MA, USA), 5 mM MgCl_2_, 10 mM HEPES pH = 7 (Sigma-Aldrich, Burlington, MA, USA), 1 mM DTT (Thermo Fisher Scientific Inc., Waltham, MA, USA), 10% digitonin (Wako, Richmond, VA, USA), 1× protease inhibitors (Pierce, Thermo Fisher Scientific Inc., Waltham, MA, USA), and 100 U/mL RNaseOut (Invitrogen, Thermo Fisher Scientific Inc., Waltham, MA, USA). Suspension was spun down at 15,000× *g* for 15 min at 4 °C. This separates organelles, membranes, and DNA from the aqueous fraction of the cells. For each IP, 100 µL of supernatant was used, along with 50 µL of washed IP bead slurry, 10 µL of 100 mM DTT, and 40 µL of 0.5 M EDTA, brought to 1 mL in an NT2 buffer (50 mM Tris pH = 7, 150 mM NaCl, 1 mM MgCl_2_, 0.05% NP40). IP tubes were incubated at 4 °C for at least 4 h on a shaker. IP input and supernatant samples were taken after incubation but before washing, final IP samples were taken after 5 washes with the NT2 buffer to confirm protein attachment. In total, 1 mL of TRIzol was added directly to washed beads, and RNA was isolated according to manufacturer protocols. In total, 10% of the amount of PLB supernatant used in the IP was used to quantify the RNA input and was isolated with TRIzol as well. Anti-FLAG-labeled IP beads were purchased from Sigma-Aldrich (Burlington, MA, USA).

### 2.9. Polymerase Chain Reaction

Reverse transcription PCR was accomplished with a Bio-Rad Laboratories T100 thermal cycler (Hercules, CA, USA). For RNA directly isolated from cells, 100 ng polyA RNA was adjusted to 10 µL with H_2_O and used with an iSCRIPT cDNA synthesis kit (Bio-Rad Laboratories, Hercules, CA, USA) and incubated with the thermal cycler according to manufacturer protocols. For RNA resulting from immunoprecipitations, 80% was used in cDNA synthesis to ensure equal amounts were used.

Real-time PCR was performed using cDNA described above, primers from Integrated DNA Technologies (See Supplemental Table S4 for primer details, San Diego, CA, USA), and Roche LightCycler products (Indianapolis, IN, USA) including FastStart DNA Green, multiwell clear plates, and the LightCycler 96 machine. cDNA was diluted 5 times with H_2_O when directly isolated from cells, and 2 times with H_2_O when resulting from immunoprecipitations.

### 2.10. RNA Sequencing

Cells from a confluent 10-cm dish were harvested with 0.05% Trypsin, quenched with media, and washed with DPBS before resuspension in 1 mL of TRIzol. After RNA isolation according to manufacturer protocols, the RNA was reprecipitated in 3 M sodium acetate (Sigma-Aldrich, Burlington, MA, USA) and 100% 2-propanol overnight at −20 °C. The next day, reprecipitation was completed, RNA was suspended in H_2_O and then quantified using Qubit 4 Fluorimeter. PolyA RNA was isolated from 100 µg of this RNA using the Poly(A)Purist MAG Kit (Invitrogen, Thermo Fisher Scientific Inc., Waltham, MA, USA) according to manufacturer protocols. PolyA RNA was quantified with the Qubit 4 Fluorimeter. In total, 200 ng of each cell line’s polyA RNA was used in the Direct cDNA Native Barcoding Nanopore protocol in preparation for sequencing using the MinION Nanopore Sequencer (Oxford Nanopore, Oxford, UK). The protocol was performed according to the manufacturer’s recommendations. Sequences were basecalled using the high accuracy basecalling setting. Passed reads were then processed using the Galaxy platform. Sequences were mapped using MinMap2 to Human Hg38 genome with the Oxford Nanopore read to reference mapping option enabled (minimap2 -x map-ont --q-occ-frac 0.01 -t 6 -a). Reads were quantified using featureCounts and differential expression determined with DESeq2. PCA analysis of individual samples showing grouping by cell line can be found in Supplemental Figure S2. The RNA sequencing data presented in this study are openly available in the GEO database with the accession number GSE217192.

### 2.11. Untargeted, Label-Free Quantitative Proteomics

Cells from five independent cell cultures for each group (biological replicates, n = 5) of cell lines with stable METTL16 expression were lifted with 1 mL of Accutase (Innovative Cell Technologies, San Diego, CA, USA) and counted using 0.4% Trypan Blue staining. Cells (~2 × 10^6^) were harvested and washed several times with DPBS and stored at −80 °C. Cell pellets were thawed on ice, resuspended in an ice cold lysis buffer (40 mM Tris-HCl, pH 8.0, 8 M urea, 40 mM NaCl, 2 mM MgCl_2_, and 1 HALT Protease Inhibitor Cocktail, Thermo Fisher Scientific Inc., Waltham, MA, USA), and subjected to 3 freeze–thaw cycles prior to sonication (30% amplitude, 15 s, 4 °C). The lysate was clarified via centrifugation (10,000× *g*, 10 min, 4 °C), and the total protein content of the clarified lysate was determined via a BCA assay (Pierce BCA Assay Kit, Thermo Fisher Scientific Inc., Waltham, MA, USA ). Equal amounts of protein (1 mg/mL) were reduced using 5 mM DTT (final concentration, 30 min, 32 °C) and then alkylated with iodoacetamide (15 mM IAM, 30 min, room temperature in the dark). Unreacted iodoacetamide was quenched with an additional 15 mM DTT. After reductive alkylation, samples were diluted to a final concentration of 1.5 M urea (40 mM Tris, pH 8.0, 30 mM NaCl, and 5 mM CaCl_2_) and digested with MS grade Trypsin overnight (1:50, total protein/Trypsin ratio, 37 °C). Samples were acidified with 0.1% formic acid and desalted via solid phase extraction (C18 SEP-PAK columns; Waters, Milford, MA, USA) Peptides were eluted (25–50% acetonitrile + 0.1% formic acid), and the resulting eluant was lyophilized overnight. Peptides were resuspended in 0.1% formic acid, and concentrations were determined via coulometric assays (Pierce Quantitative Peptide Assay, Thermo Fisher Scientific Inc., Waltham, MA, USA ). Concentrations were adjusted to 0.25 mg/mL with 0.1% formic acid.

MS-based proteomic analysis was performed on an UltiMate 3000 RSLCnano system (Thermo Fisher Scientific Inc., Waltham, MA, USA) coupled to a Q Exactive Plus Hybrid Orbitrap mass Spectrometer (Thermo Fisher Scientific Inc., Waltham, MA, USA) via nanoelectrospray ionization as previously described [77]. Peptides were separated using an effective linear gradient of 10–40% acetonitrile (0.1% formic acid) over 120 min. For data dependent acquisition, MS spectra were acquired in the positive mode. MS1 was performed at a resolution of 70,000 with an AGC target of 2 × 10^6^ ions and a maximum injection time of 100 ms. Data dependent acquisition was used to collect MS2 spectra on the top 15 most abundant precursor ions with a charge >1 and an isolation window of 1.5 *m*/*z* and fixed first mass of 140 *m*/*z*; a normalized collision energy for MS2 scans was 27. MS2 spectra were acquired at a 17,500 resolution, maximum injection time of 50 ms, and AGC target of 1 × 10^5^.

The proteomics data presented in this study are openly available in the ProteomeXchange Consortium via the PRIDE partner repository [78] with the dataset identifier PXD042242.

### 2.12. Proteomics Data Analysis

Proteome Discover 2.5 was used for the processing of raw data files and protein identification. Default search parameters included Met oxidation (+15.99492 Da) as a variable modification and Cys carbiomidomethylation (+57.02146 Da) as a fixed modification. Data were searched against the Uniprot Homo Sapiens reference proteome (UP000005640, downloaded 3 March 2022) supplemented with additional sequences for the wild-type, PP185/186AA, N-terminal, and Δ-Linker METTL16 mutant and CRISPR proteins. Precursor ion *m*/*z* tolerance was ±10 ppm, and fragment ion *m*/*z* tolerance ±0.6 Da with three missed cleavages allowed. The search results were filtered by a 1% false discovery rate at the peptide level. Strict parsimony was used to group peptides to proteins. Label-free quantification was carried out using MS1 precursor intensity. Intensities were normalized to both the average intensities and the distribution width of the intensities within the sample. Relative abundances for low sampling proteins were determined via imputation [79]. Assuming a normally distributed population and unequal variance between the control and experimental values, we used Welch’s two sample two-tailed *t*-test in GraphPad Prism and considered all proteins with a *p*-value ≤ 0.05 to be statistically significant.

### 2.13. Functional Annotations

Functional annotation and pathway enrichment analysis was performed using the PANTHER Overrepresentation Test [80] (Released 13 October 2022), the GO ontology database [81] (Released 1 July 2022), and the GO molecular function complete annotation set. Depleted pathways, e.g., negative enrichment, were calculated from depleted protein abundances compared to the wild-type cell line, whereas enriched pathways were determined from increased protein abundances. Proteins with a minimum of 3-fold change in relative abundance compared to HEK293 (wild-type) or the wild-type cell line were used for analysis. Fisher’s exact test with FDR correction with the Bonferroni correction (*p*-value < 0.05) was used to establish significance.

### 2.14. EdU Flow Cytometry Assay

Once cell lines were established and expressions verified, cells were plated at 0.5 × 10^6^ cells in a 10-cm dish and allowed to attach overnight. The next day, 10 µL of 10 mM EdU (Click-iT Plus EdU Alexa Fluor 488 Flow Cytometry Assay Kit, Invitrogen, Thermo Fisher Scientific Inc., Waltham, MA, USA) was added to each culture and incubated at 37 °C for 1 h, after which the media were replaced with fresh media and incubated for another 3 h. Cells were then lifted with 1 mL 0.05% Trypsin, quenched with media, collected, and washed once with 1% BSA (Fisher Scientific Inc., Hampton, NH, USA) in DPBS. Cells were then fixed and stained using the Click-iT kit contents and DAPI (Invitrogen, Thermo Fisher Scientific Inc., Waltham, MA, USA) according to manufacturer protocols. Cells were analyzed for fluorescence with the Cytek Aurora Flow Cytometer using SpectroFlo and FlowLogic software version 8.4 for analysis.

### 2.15. Proliferation Assay

Approximately 2.5 × 10^5^ cells were plated in to each well of a 6-well plate. At 24, 48, and 72 h after plating, cells were lifted with 0.05% Trypsin and counted via hemocytometer and/or a LUNA-FL automated cell counter (Logos Biosystems, Anyang, Kyonggi-do, South Korea). Media were refreshed on remaining cells every 24 h to avoid nutrient/additive deficiency during the time frame of the experiment and to allow for constant proliferation.

## 3. Results

To observe the responsibilities of each METTL16 protein domain, we created transgenic HEK293 cell lines expressing a METTL16 protein mutated in one of three domains (Figure 1A). The mutations included an N-terminal RNA-binding domain mutant (described previously by Mendel et al., 2018 [76]), a methylation mutant (PP185/186AA, described previously by Pendleton et al. 2017 [35]), a C-terminal RNA-binding mutant (in which the VCR linker region was deleted), and a wild-type control. The sequence also contained a silent mutation, rendering it resistant to a CRISPR-Cas9 gRNA sequence to be used later, and a Myc-FLAG tag at the C-terminus. These mutations were introduced via site-directed mutagenesis (primers can be found in Supplemental Table S2) and confirmed with Sanger sequencing. Stable, clonal lines of each METTL16 mutation, as well as a wild-type overexpression line were produced with antibiotic selection and then colony selection with expression verified via Western blotting.

The RNA-binding abilities of all the METTL16 variants were tested using FLAG immunoprecipitation. As expected, the N-terminal RNA-binding mutant showed very little binding ability; however, the methylation mutant (PP185/186AA) also showed stunted RNA binding. We continued with this mutant but also produced a second methylation mutant (N184A, previously described by Doxtader et al. 2018 [64]) which showed comparable RNA-binding ability to the wild-type protein. Interestingly, deletion of the VCR linker region in our C-terminal RNA-binding domain mutant resulted in increased RNA-binding ability over the wild-type protein.

After confirming the expression and RNA-binding ability of METTL16 in each of the clonal overexpression lines, we introduced a METTL16 CRISPR-Cas9 construct targeting exon 2 of the endogenous METTL16 locus, selected for expression via puromycin, and generated clonal lines of each METTL16 mutant, as well as the wild-type overexpression line. Due to the strength of the plasmid’s promoter, a higher expression level compared to the endogenous METTL16 was observed (Figure 1B). Clones were screened for removal of endogenous METTL16 but continued expression of the exogenous METTL16. Due to the Myc-FLAG tag on the exogenous METTL16, separation of the two METTL16 species was achieved with a long gel electrophoresis run and subsequent Western blotting (Supplemental Figure S1). The Western blot confirmed there was no detectable endogenous METTL16 protein expression in any of the METTL16 transgenic cell lines. Of note, during the continued culturing of these mutant cell lines, the first methylation mutant (PP185/186AA) line lost all detectable METTL16 expression (Figure 1B). It is our hypothesis that the double proline–double alanine mutation in METTL16 is detrimental to both the methylation ability and the appropriate physical structure of the protein. If true, this mutant version may serve no purpose or even have adverse effects on the cells, leading them to eventually silence expression. We continued to include this cell line in the experiments; however, we interpreted the results of this cell line as if it was a METTL16 null line. Of interest, MAT2A protein levels showed little change between the stable mutant METTL16 cell lines (Figure 1B).

Once exogenous METTL16 expression was verified, we again tested the RNA-binding ability of the mutants to ensure CRISPR-Cas9 exposure did not alter the intended METTL16 mutation. For this we used METTL16 immunoprecipitation in comparison to the wild-type exogenous METTL16 line as well as the starting HEK293 line (data not shown). Methylation ability was previously shown to be ablated with the PP185/186AA, N184A, and N-terminal RNA-binding METTL16 mutant proteins [35,59,76] via in vitro methylation assays and was not further investigated here.

Previously, we had shown that endogenous and exogenous wild-type METTL16 was predominantly cytoplasmic in a number of cell lines [82]. To determine the effect of METTL16 mutation on cellular localization we used biochemical fractionation Western blots. Antibodies for nuclear lamina protein Lamin B and cytoplasmic protein lactate dehydrogenase were used to confirm fractions of cells were pure. As shown in Figure 1C, all mutated METTL16 proteins had similar localization as endogenous METTL16, with most located in the cytoplasm. Thus, it appears that mutating METTL16 in these ways does not affect its cellular distribution. Lastly, we investigated the half-life of the exogenous METTL16 proteins using cycloheximide to block translation (Figure 1D).

**Figure 2:**
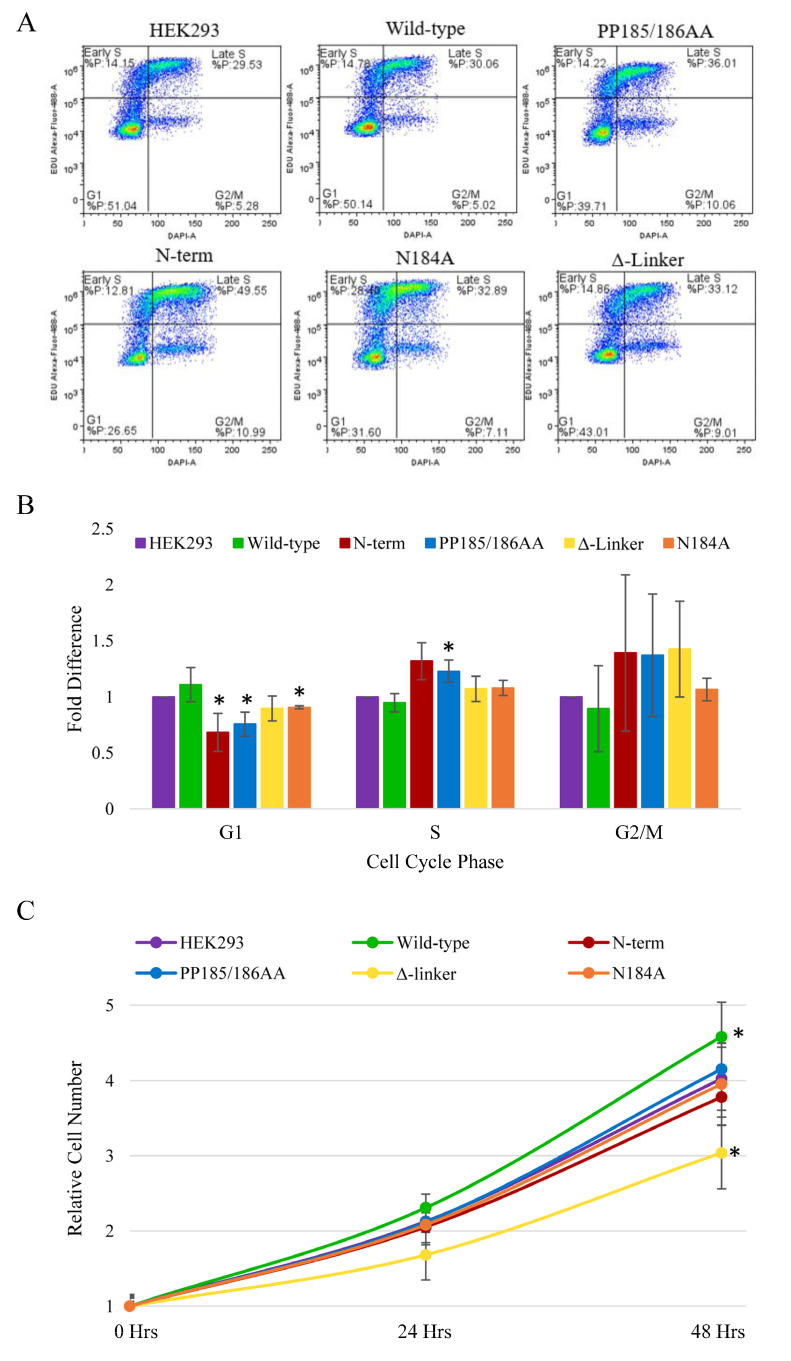
EdU Assays with HEK293 and HEK293 METTL16 mutant CRISPR clonal cell lines used in this study. “Wild-type” is the non-mutated exogenous METTL16 line, “PP185/186AA” is the PP185/186 methylation mutant exogenous METTL16 line, “N-term” is the N-terminal RNA-binding mutant exogenous METTL16, “Δ-Linker” is the C-terminal RNA-binding mutant exogenous METTL16 line, and “N184A” is the N184A methylation mutant exogenous METTL16 line. (**A**) Representative flow cytometry plots from each cell line. (**B**) Analysis of EdU assay results from each cell line. Percentage of HEK293 cells in each phase shown was normalized to 1. Percentages of M16 mutant CRISPR clonal cells were normalized to HEK293. (**C**) Cell proliferation over 48 h (Hrs) for each cell line normalized to the zero-time point. *p*-value < 0.05 indicated with *. Representative of 3–7 experiments.

Again, we determined the half-life of the METTL16 protein in these clones closely mimicked the half-life of the HEK293 endogenous METTL16, which was shown to be more than 16 h. Therefore, we have shown METTL16 to be a long-lived nuclear and cytoplasmic protein, and that these characteristics are not substantially changed when either the methylation or RNA-binding activity of the protein is altered. Given the recent correlations cited between METTL16 expression and cancer progression, we performed an EdU assay with these clonal lines to investigate changes in cell cycle (Figure 2). Using this method, we were able to quantify the number of cells in the G1, S, or G2/M phase of the cell cycle (Figure 2A). To quantify the effect, the percentage of HEK293 cells in each cell cycle phase was normalized to 1 and then the relative change in each METTL16 transgenic cell line was calculated (Figure 2B). There was no significant difference in G1, S, or G2 phase occupancy times between the starting HEK293 cell line and the transgenic wild-type METTL16 cell line. However, the METTL16 null line (PP185/186AA) with no detectable METTL16 expression and the N-terminal RNA-binding mutant had a lower occupancy time in the G1 phase and more occupancy in the S phase compared to HEK293 cells (Figure 2B). This suggests these two cell lines proliferate slower than the starting cell line and the wild-type transgenic control. The C-terminal and N184A mutant transgenic lines showed no significant difference in cell cycle phase occupancy. SubG1 analysis indicated no significant cell death in any of the cell lines. Given that the G2 phase is shorter than G1 and S, and that the assay detects DNA content, we were unable to determine any significant differences during that cell cycle phase using this method.

Given that the changes to cell cycle occupancy were subtle (yet significant), we also determined the relative proliferation of each cell line over a 48-h time course (Figure 2C). Overexpression of the wild-type METTL16 increased cellular proliferation as compared to the parental HEK293 cell line, with the effect becoming significant at 48 h. The only METTL16 mutation to show a significant effect on proliferation was the deletion of the VCR linker region (Δ-linker), which again showed a significant effect compared to the HEK293 cells at 48 h; it was significantly different from the wild-type METTL16 line at both 24 and 48 h.

**Figure 3:**
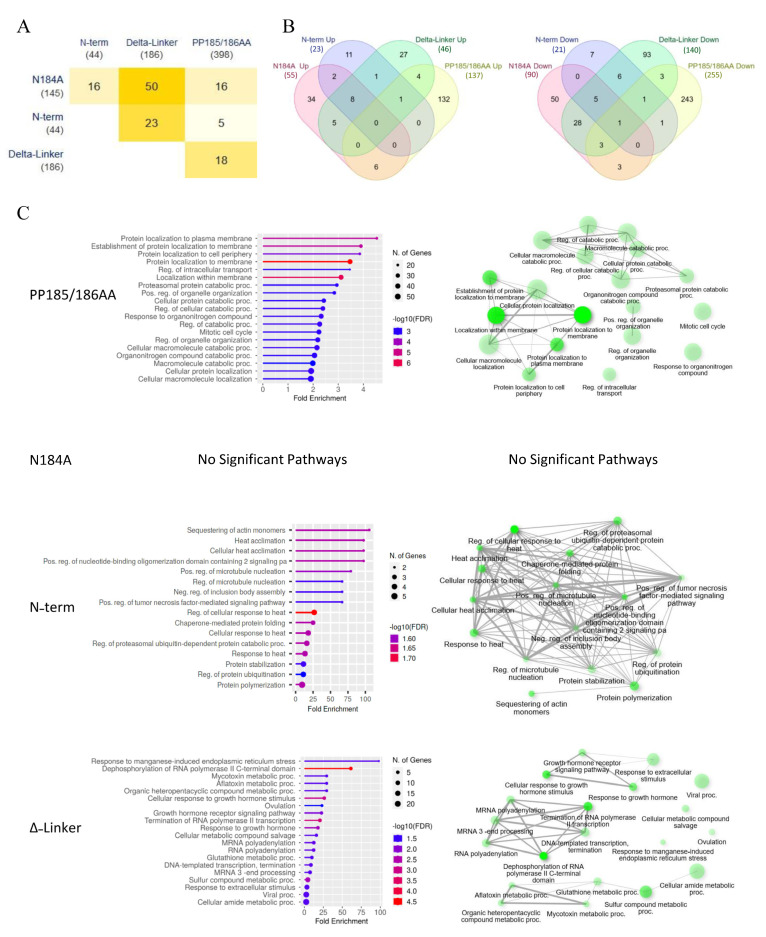
RNA sequencing results of METTL16 transgenic cell lines produced in this study. (**A**) Overlap of all genes significantly different from WT METTL16 line. (**B**) Overlap of genes significantly increased (**left**) or decreased (**right**) as compared to WT METTL16 line. Numbers in (parenthesis) are total number of significant genes in each mutant. (**C**) Gene set enrichment analysis of the genes significantly different from WT METTL16 line. (**Left**): Enrichment charts of the significant gene sets ranked by fold enrichment. (**Right**): Network maps of significantly altered pathways. All analysis was performed with ShinyGo 0.76.2.

**Figure 4:**
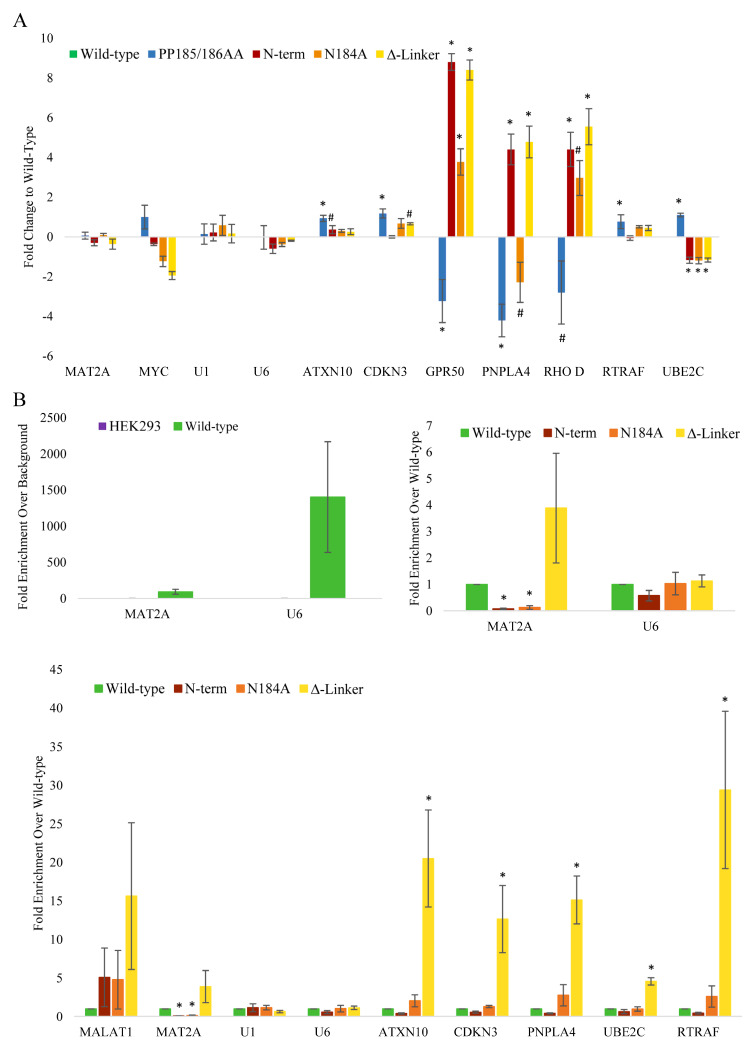
Effect of METTL16 mutations on mRNA expression and binding. (**A**) Expression results from HEK293 METTL16 mutant CRISPR clonal lines. RNA expression from real-time PCR analysis of each cell line with all normalized to wild-type transgenic METTL16 line. Representative of 3–4 experiments; error bars display SEM, # indicates *p* < 0.1, * indicates *p* < 0.05. (**B**) FLAG RNA immunoprecipitation results from each METTL16 mutant CRISPR clonal line used in this study. “Wild-type” is the non-mutated exogenous METTL16 line, “PP185/186AA” is the PP185/186 methylation mutant exogenous METTL16 line, “N-term” is the N-terminal RNA-binding mutant exogenous METTL16, “Δ-Linker” is the C-terminal RNA-binding mutant exogenous METTL16 line, and “N184A” is the N184A methylation mutant exogenous METTL16 line. Representative of 3 experiments; error bars display SEM, * indicates *p* < 0.05.

To further investigate METTL16 mutation-driven changes, RNA sequencing and untargeted proteomics analysis were performed. For RNA sequencing, polyA RNA was extracted from the clones and sequenced as unamplified, barcoded cDNA using the Oxford Nanopore MinION Sequencer. Interestingly, mutating the various domains of METTL16 only resulted in significant changes to about 50–400 transcripts compared to the transgenic wild-type cell line (Figure 3A, Supplemental Dataset S1). In addition, while some of the gene expression changes were shared amongst the mutants, the majority were unique to a given mutant line (Figure 3B). Gene set enrichment analysis of the significantly altered genes was then used to identify unique subsets that were altered in the mutant lines (Figure 3C). As might be expected, the METTL16 null line (PP185/186AA) showed the greatest effect of all four mutant lines, with almost 400 genes altered (Figure 3A). The major pathways altered in the null mutant were involved in protein localization, catabolic processes, organelle organization, and mitotic cell cycle (Figure 3C) Surprisingly, the methylation mutant (N184A) showed the smallest effect with only 44 genes altered and no significant GO pathways identified. This suggests that METTL16’s methylation activity may only be a small part of its overall cellular activity. The two RNA-binding mutants both had a moderate effect on gene expression but affected vastly different pathways (Figure 3C), suggesting that each domain plays a distinct role in METTL16’s activity. Expression changes of a select group of mRNAs were confirmed via real-time PCR with results normalized to the wild-type METTL16 transgenic line (Figure 4A). The N-terminal and C-terminal RNA-binding METTL16 mutant lines generally showed the same trend of expression change in those RNAs affected. We did not see RNA expression changes in U1 snRNA (our negative control), MAT2A mRNA, or U6 snRNA. There were several RNAs whose expression significantly changed in all four METTL16 transgenic lines, but the expression varied among the different lines, again suggesting differing effects of the various mutants.

To determine whether the mRNA expression changes could be linked to direct interaction with METTL16, we performed FLAG immunoprecipitations followed by real-time PCR normalized to the wild-type METTL16 transgenic line. As the best characterized METTL16 targets, MAT2A mRNA and U6 snRNA were significantly bound by the wild-type transgenic METTL16 as expected (Figure 4B). Mutation of the N-terminal RNA-binding domain decreased binding to MAT2A mRNA significantly, as did the N184A methylation mutant (Figure 4). Intriguingly, the C-terminal RNA-binding mutant bound MAT2A mRNA more than the wild-type METTL16. This binding trend was also observed amongst most of the probed RNAs shown in Figure 4, in which the N-terminal RNA-binding METTL16 mutant bound less of the RNA, the N184A methylation METTL16 mutant showed similar binding, and the C-terminal RNA-binding METTL16 mutant showed higher binding. In fact, the C-terminal RNA-binding METTL16 mutant showed significantly higher binding in most of the RNAs probed. Despite differences in binding to the mRNAs tested, for all transgenic lines METTL16 bound the U6 snRNA in similar amounts (Figure 4).

**Figure 5:**
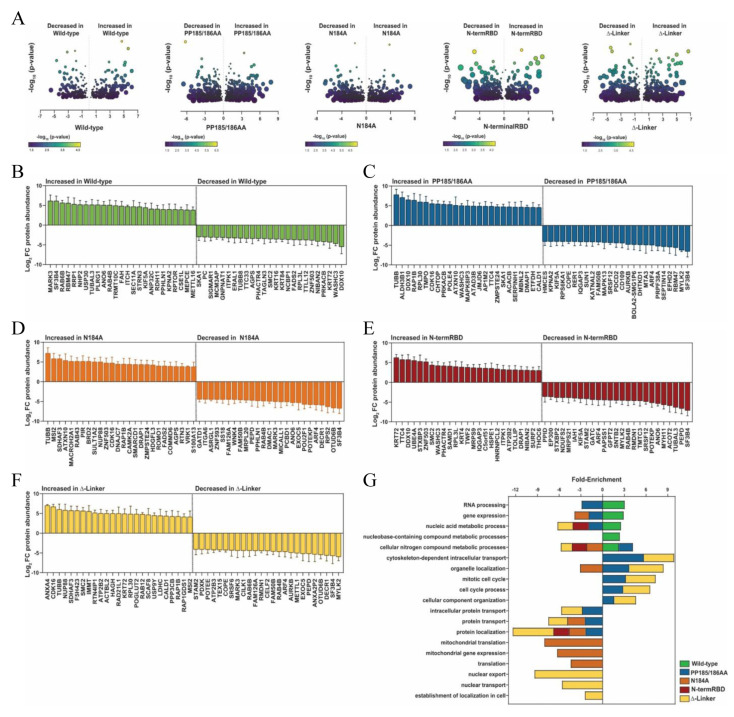
Proteomic analysis of each cell line. (**A**) Volcano plots of −log10 (*p*-value) vs the log2 fold changes in protein abundances for statistically significant proteins (n = 5 biological replicates, Welch’s unpaired *t*-test, *p* < 0.05). Symbols are colored by −log10 (*p*-values) and symbol sizes are relative to the SEM of the log2 fold change. Protein abundances for the wild-type were initially compared to HEK293 cells. Protein abundances for all other cell lines were subsequently compared to the wild-type cell line. (**B**–**F**) Plots of log2 fold changes in protein abundances for the top 25 proteins exhibiting the greatest increase and decrease in each cell line. Log2 fold changes in protein abundance for the wild-type, non-mutated exogenous METTL16 cell line compared to HEK293 controls (**B**) or to the wild-type cell line (**C**–**F**) are shown as mean ± SEM from n = 5 biological replicates for statistically significant differences (Welch’s unpaired *t*-tests, *p* < 0.05). (**G**) Go Enrichment Pathway analysis. Stacked plot showing select results from gene pathway enrichment analysis using PANTHER Classification System [78] and proteins exhibiting at least a 3-fold change in protein abundances when compared to HEK293 (wild-type) or the wild-type cell line. Positive fold enrichment arises from increased proteins abundances, negative enrichment arises from decreased protein abundances compared to the wild-type cell line.

To assess global changes in whole-cell protein abundances, we performed proteomics analysis on all five cell lines (Supplemental Dataset S2). Overall, we identified 300–700 statistically significant altered proteins compared to the wild-type exogenous METTL16 clone, depending on the mutant (Figure 5). Volcano plots of all proteins identified with high confidence are displayed in Figure 5A, with significant differences in expression between the two cell lines compared and located above the dotted horizontal line. As with other experiments in this study, we compared the proteins identified in HEK293 cells against those identified in the wild-type METTL16 transgenic cell line, and the identified proteins in the mutant METTL16 transgenic line were compared to the wild-type METTL16 transgenic line proteins. With these results, we also explored the top 25 proteins that increased and the top 25 proteins that decreased in expression in Figure 5B–F. Gene pathway enrichment analysis using the PANTHER Classification System [80] was then performed on all proteins exhibiting a three-fold or greater change in protein abundance as compared to the respective control line. Again, similar to the RNA expression changes, about 50% of the significant protein changes seen in each mutant cell line were unique to that cell line, with varying amounts of overlap with the others (Figure 3C and Figure 5), suggesting differing effects of the mutants on METTL16’s cellular function. Interestingly, there was very little overlap between significant RNA and protein changes within each cell line (Supplemental Figure S3).

## 4. Discussion

After our previous published study in 2020 showing METTL16 protein was localized to both the cytoplasm and the nucleus, and other publications showing lack of complete characterization of the protein’s domains, we hypothesized METTL16, in addition to RNA methylation, had another role related to its RNA-binding ability [82]. To investigate this, we conceptualized and produced cell lines with one of the METTL16 protein’s domains mutated to observe the impact of the partial loss of function on the cell, including the N-terminal RNA-binding domain, the methyltransferase domain, and the VCR RNA-binding domain. Because METTL16 was thought to be essential for all cells at the time of project conception, we opted to first introduce the mutated METTL16 into cells before removing the endogenous METTL16 from the genome to ease the cells into the transition. Given our and others’ success in mutating/removing METTL16 [34,44], it may be that METTL16 is only essential for certain cell types, such as stem cells, certain embryonic cells, or cells not yet included in METTL16 studies.

From our mutated METTL16 cell lines, we have determined first that METTL16’s methylation ability appears to be dispensable for cell survival. Given that m6A methylation is a further fine-tuning of the RNA’s processing or half-life, it is probable that there are other modes of regulation in place that are more influential on the RNA affected by METTL16. This is not to say there are no consequences to removing METTL16’s methylation ability. We saw widespread protein expression changes with our METTL16 N184A mutant cell line which suggests that methylation does indeed play a role in METTL16’s function, perhaps by regulating translation of a subset of proteins.

Secondly, we determined METTL16’s RNA-binding ability is reliant on both the N-terminal and C-terminal RNA-binding domains. Mutation of the METTL16 N-terminal RNA-binding domain resulted in lower relative amounts of immunoprecipitated RNA but did not completely abrogate binding. This mutant was adapted from Mendel et al., who showed that mutation of the N-terminal RNA-binding domain completely abrogated binding to the MAT2A hairpin 1 when the C-terminal domain of METTL16 was not present. Interestingly, when we deleted the linker region between the VCRs of the C-terminal RNA-binding domain, it actually increased the RNA-binding efficiency of METTL16. Almost every RNA for which we probed in the immunoprecipitation studies showed increased binding to the C-terminal transgenic METTL16 compared to the wild-type transgenic METTL16. We speculate that removal of the linker region between the vertebrate conserved regions in this mutant slowed and/or limited the range of motion of the vertebrate conserved region domain as a whole and therefore allowed for stronger or longer binding. The region deleted is not evolutionarily conserved and is a disordered region [67]. Therefore, it is probable that this disordered region is vital to attenuating the RNA binding that the rest of the domain does. It is interesting to note that METTL16 has been implicated in recruiting RNA to translation machinery [44]. Further studies will determine which of these RNA-binding domains (if not both) is responsible for the aid in translation.

Because we suspected METTL16 to bind and/or influence more RNAs than those currently published, we searched for changes in protein and RNA expression in the clonal lines we produced. We determined more extensive changes in protein expressions compared to RNA expressions but found little correlation between them (Supplemental Figure S3). This could be because our RNA sequencing analysis did not probe for splicing changes among the RNAs identified. It could also be due to the recently cited role of METTL16 in translation [44]. There were some similarities among the proteins whose expression changed among the clonal lines; however, there were even more differences, indicating recruitment to the translation machinery is not the only reason for the noted changes. For instance, we saw β-tubulin protein increased in all mutant lines compared to the wild-type transgenic line. We also identified nucleic acid metabolic process, cellular nitrogen compound metabolic processes, and protein localization pathways affected in three out of four mutant lines each compared to the wild-type transgenic line. However, we saw cytoskeleton-dependent intracellular transport, mitotic cell cycle, cell cycle process, cellular component organization, and intracellular protein transport pathways affected in only two (PP185/186AA; delta-linker) of the four mutants, and multiple pathways affected in only one mutant.

From our RNA expression and immunoprecipitation studies, we have identified ATXN10, CDKN3, PNPLA4, and RTRAF as new potential RNA targets of METTL16. From our analysis, we have determined the RNAs affected by METTL16 alteration belong in one of three groups. Group 1 contains RNAs that bind METTL16 but do not reflect an expression change when METTL16 protein becomes mutated. Among this group is MAT2A mRNA and U6 snRNA. Previous studies have shown the effects of METTL16 manipulation on MAT2A RNA and protein levels, which we did not observe. The lack of change in expression is not necessarily indicative of a lack of effect on the RNA; for instance, U6 snRNA expression is not expected to change, and MAT2A mRNA is more likely to show splicing changes, which we did not investigate in this study. Other RNAs that fall into this group are likely to be imperative to cell survival, such as the two listed. Group 2 contains RNAs that show expression changes but do not seem to bind METTL16. These RNA expressions are most likely changing as an indirect or secondary effect of true METTL16 target changes. Group 3 contains RNAs that both bind METTL16 and show RNA expression changes. Among this group is ATXN10 and PNPLA4 mRNAs. METTL16 may have more than one direct effect on these RNAs, or it is possible they are both directly and indirectly affected by METTL16. Even though the RNAs of groups 1 and 3 are of the most interest, RNAs in group 2 aid in the cell-wide effects observed by altering METTL16.

Regardless of the exact mechanisms METTL16 uses to produce the changes we have observed, we have shown that it significantly affects the cell cycle timing. Our EdU assay results show our transgenic METTL16 cell lines spend more time in S phase if METTL16 is mutated in certain domains. Furthermore, we determined CDKN3 to be an RNA-binding target of METTL16, which is well known to affect the G1/S phase transition of the cell cycle. Future studies will determine the effect this binding has on CDKN3 RNA, whether METTL16 methylates it, and its subsequent protein expression.

Interestingly, while expression of the mutant METTL16 showed effects on cell cycle progression, it often did not correlate to significant effects on cellular proliferation. However, it does appear that overexpression of wild-type METTL16 did increase proliferation as compared to the parental HEK293T line. This is intriguing as METTL16 overexpression has been implicated in a number of cancer types [43,44,45,47,48,49,50,51,83]. Furthermore, deletion of the VCR linker region appears to inhibit METTL16’s ability to increase proliferation and perhaps even interferes with its normal cellular function. Future studies into the role of METTL16 in cancer may provide insight into this mechanism, and better understanding of the role of the VCR linker region may provide direction toward therapeutics for METTL16-driven tumors.

Interest in METTL16’s cellular functions continues to grow and more data is becoming available, but at the time of this publication, molecular-based knowledge about METTL16 is still incomplete. This study has added to that knowledge by further revealing RNAs, proteins, and cell processes affected by the METTL16 protein. However, more studies are needed for a better understanding of METTL16. The expression of METTL16 mutants in HEK293 cells under basal cell conditions in this study does limit the view of METTL16’s full potential impact on cell processes. Expressing these mutants in different cell types and subjecting them to environmental and cellular stressors could further reveal how METTL16 contributes to, or combats, cell responses in different states more applicable to human physiology. Once the foundational work is completed, disease-based studies can then be properly performed and interpreted.

Overall, the METTL16 mutant cell lines produced for this publication will provide insight into disease states where METTL16 has been documented as mutated or dysregulated. Most diseases associated with METTL16 are currently listed as due to an over- or under-expression of the protein. There is one documented type of large intestinal cancer which has a mutation (in the methyltransferase domain) that abolishes METTL16’s methylation activity [64]. More in-depth analysis of the METTL16 protein in these types of studies may reveal a pattern of mutation correlating with expression. The investigation of mutated METTL16 qualities and cellular outcomes such as those found in this study will link the functionality of METTL16 with its consequences in disease.

## Data Availability

The RNA sequencing data presented in this study are openly available in the GEO database with the accession number GSE217192. The proteomics data presented in this study are openly available in the ProteomeXchange Consortium via the PRIDE partner repository [82] with the dataset identifier PXD042242.

## Supplementary Materials

The following supporting information can be downloaded at: https://www.mdpi.com/article/10.3390/cimb45070346/s1, Supplemental Figure S1: Western blot of clonal METTL16 expression. Supplemental Figure S2: PCA analysis of deep sequencing. Supplemental Figure S3: Correlation between transcriptomic and proteomic data. Supplemental Figure S4: Original Western blots. Supplemental Dataset S1: Deep sequencing mRNA fold change. Supplemental Dataset S2: Mass Spectrometry protein fold change; Supplemental Tables S1–S4: Antibodies, plasmids, and primers used in this study.
