# Supplementary material for: RNA Methyltransferase METTL16’s Protein Domains Have Differential Functional Effects on Cell Processes
Source: Curr Issues Mol Biol. 2023 Jun 29;45(7):5460–80. doi: 10.3390/cimb45070346 (PMC10378215; doi:10.3390/cimb45070346)
Supplement: Supplementary file 1 [file cimb-45-00346-s001.zip › Supplemental Figure 1_Western blot of clonal METTL16 expression.pdf]

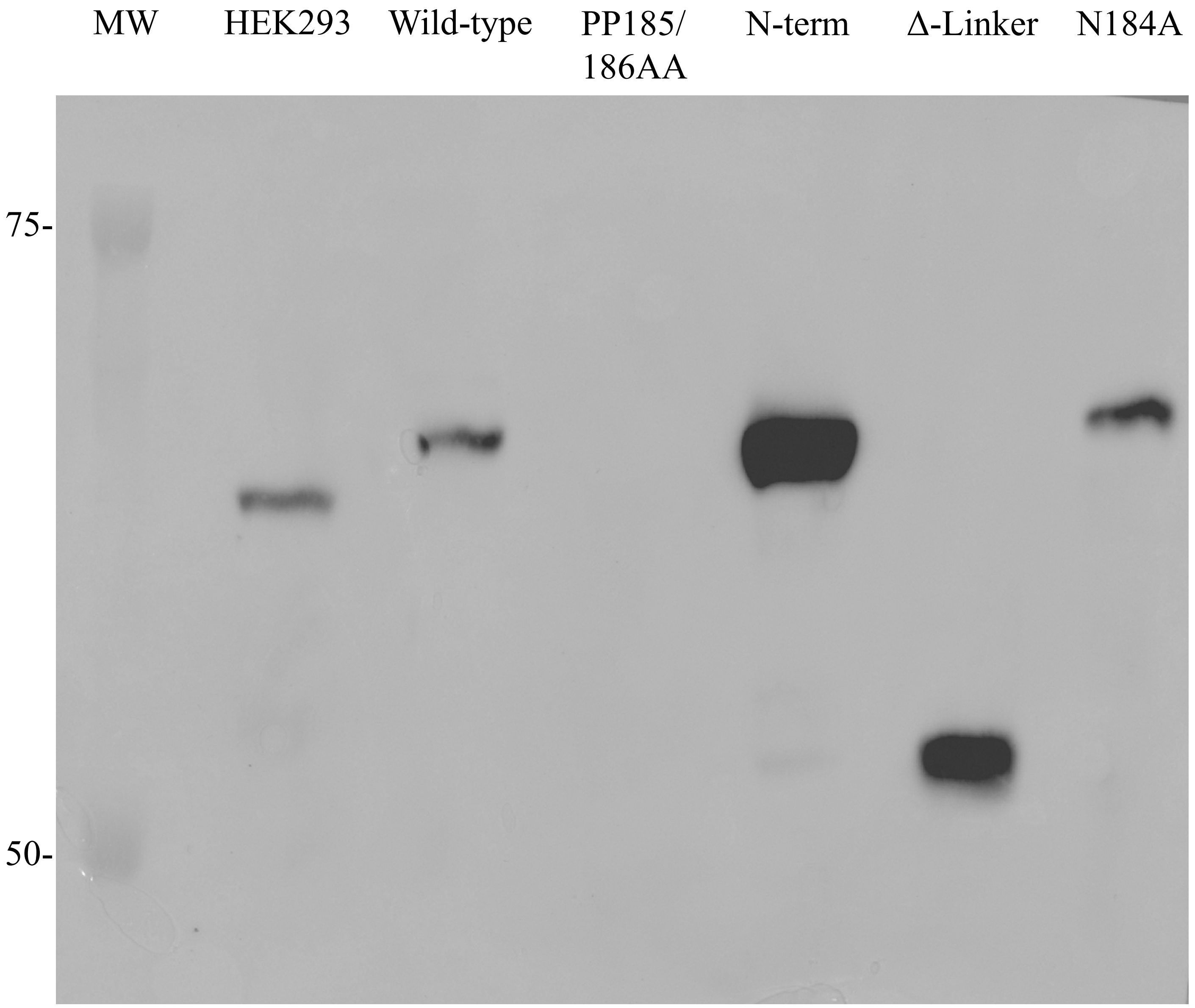

Supplemental Figure 1: Western blot of HEK293 transgenic METTL16 mutant cell lines with endogenous METTL16 removed via CRISPR-Cas9 probed with METTL16 antibody. Large separation polyacrylamide gel shows the difference in molecular weight (MW) between endogenous METTL16 found in HEK293 cells, and transgenic mutant METTL16 proteins found in the clonal cell lines generated for this study. Mutant PP185/186AA cell line shows no expression of METTL16 indicating loss during clonal selection/growth. Molecular weight markers of 50 and 75 Kd are also shown for size reference.
