# Supplementary material for: RNA Methyltransferase METTL16’s Protein Domains Have Differential Functional Effects on Cell Processes
Source: Curr Issues Mol Biol. 2023 Jun 29;45(7):5460–80. doi: 10.3390/cimb45070346 (PMC10378215; doi:10.3390/cimb45070346)
Supplement: Supplementary file 1 [file cimb-45-00346-s001.zip › Supplemental Figure 2_PCA Analysis.pdf]

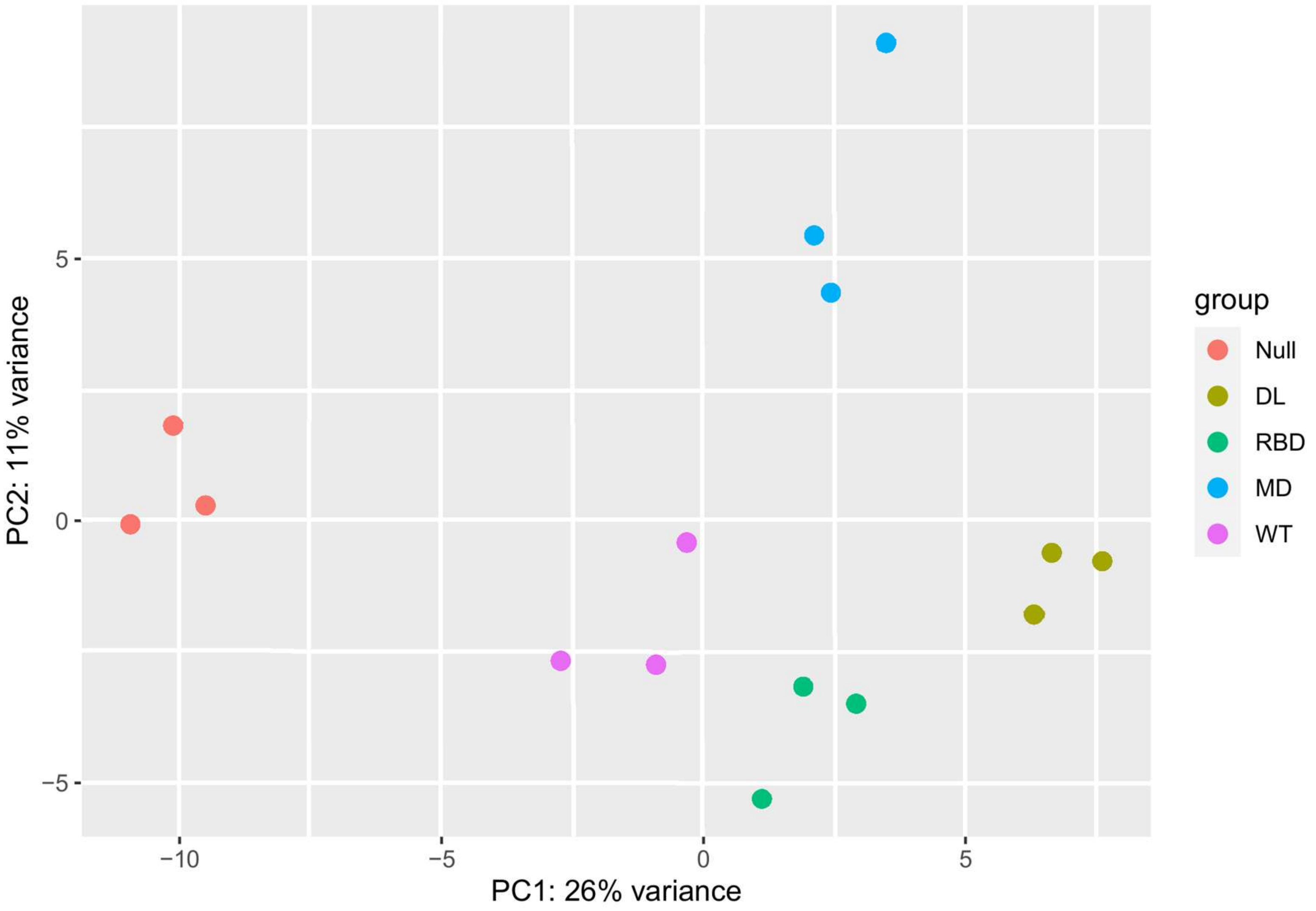

Supplemental Figure 2: Principal Component Analysis of deep sequencing data. Each colored circle represents individual sample. Lines tested included Null: PP185/186AA mutant; DL: Delta Linker mutant; RBD: N-terminal RNA Binding Domain mutant; MD: N184A methylation domain mutant; WT: Wild type METTL16.
