# Supplementary material for: RNA Methyltransferase METTL16’s Protein Domains Have Differential Functional Effects on Cell Processes
Source: Curr Issues Mol Biol. 2023 Jun 29;45(7):5460–80. doi: 10.3390/cimb45070346 (PMC10378215; doi:10.3390/cimb45070346)
Supplement: Supplementary file 1 [file cimb-45-00346-s001.zip › Supplemental Figure 3_Sequencing_Proteomics_correlation.pdf]

Supplemental Figure 3

	N-term RNA (44)	Delta-linker RNA (186)	PP185/186AA RNA (398)	N184A PTN (569)	N-term PTN (324)	Delta-linker PTN (498)	PP185/186AA PTN (681)
N184 RNA (145)	16<br>0.0925	50<br>0.1779	16<br>0.0304	12<br>0.0171	5<br>0.0108	7<br>0.0110	12<br>0.0147
N-term RNA (44)		23<br>0.1111	5<br>0.0114	2<br>0.0033	2<br>0.0055	2<br>0.0037	3<br>0.0042
Delta-linker RNA (186)			18<br>0.0318	12<br>0.0162	3<br>0.0059	13<br>0.0194	13<br>0.0152
PP185/186AA RNA (398)				26<br>0.0276	11<br>0.0155	28<br>0.0323	40<br>0.0385
N184A PTN (569)					113<br>0.1449	164<br>0.1816	175<br>0.1628
N-term PTN (324)						113<br>0.1594	123<br>0.1395
Delta-linker PTN (498)							188<br>0.1897

0 Jaccard index 1

Supplemental Figure 3: Correlation Matrix of Deep Sequencing and Proteomics data. Correlation of significantly changed genes (number in parenthesis) from either deep sequencing (RNA) or proteomics (PTN) analysis. Top number in matrix box indicates number of shared gene symbols while bottom number is the Jaccard similarity coefficient.
