# Supplementary material for: RNA Methyltransferase METTL16’s Protein Domains Have Differential Functional Effects on Cell Processes
Source: Curr Issues Mol Biol. 2023 Jun 29;45(7):5460–80. doi: 10.3390/cimb45070346 (PMC10378215; doi:10.3390/cimb45070346)

Figure 1B: METTL16

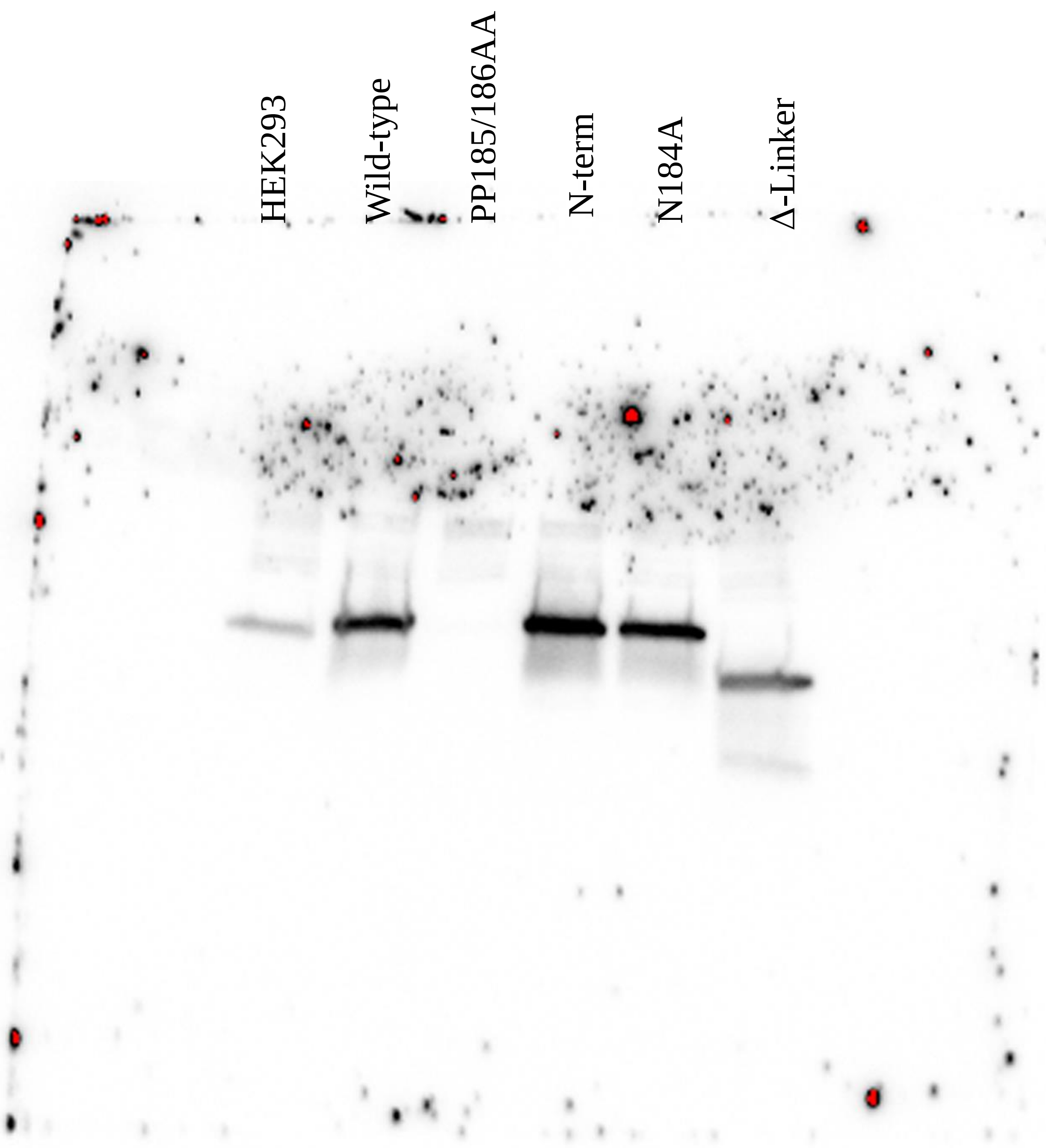

Figure 1B: γ-tubulin

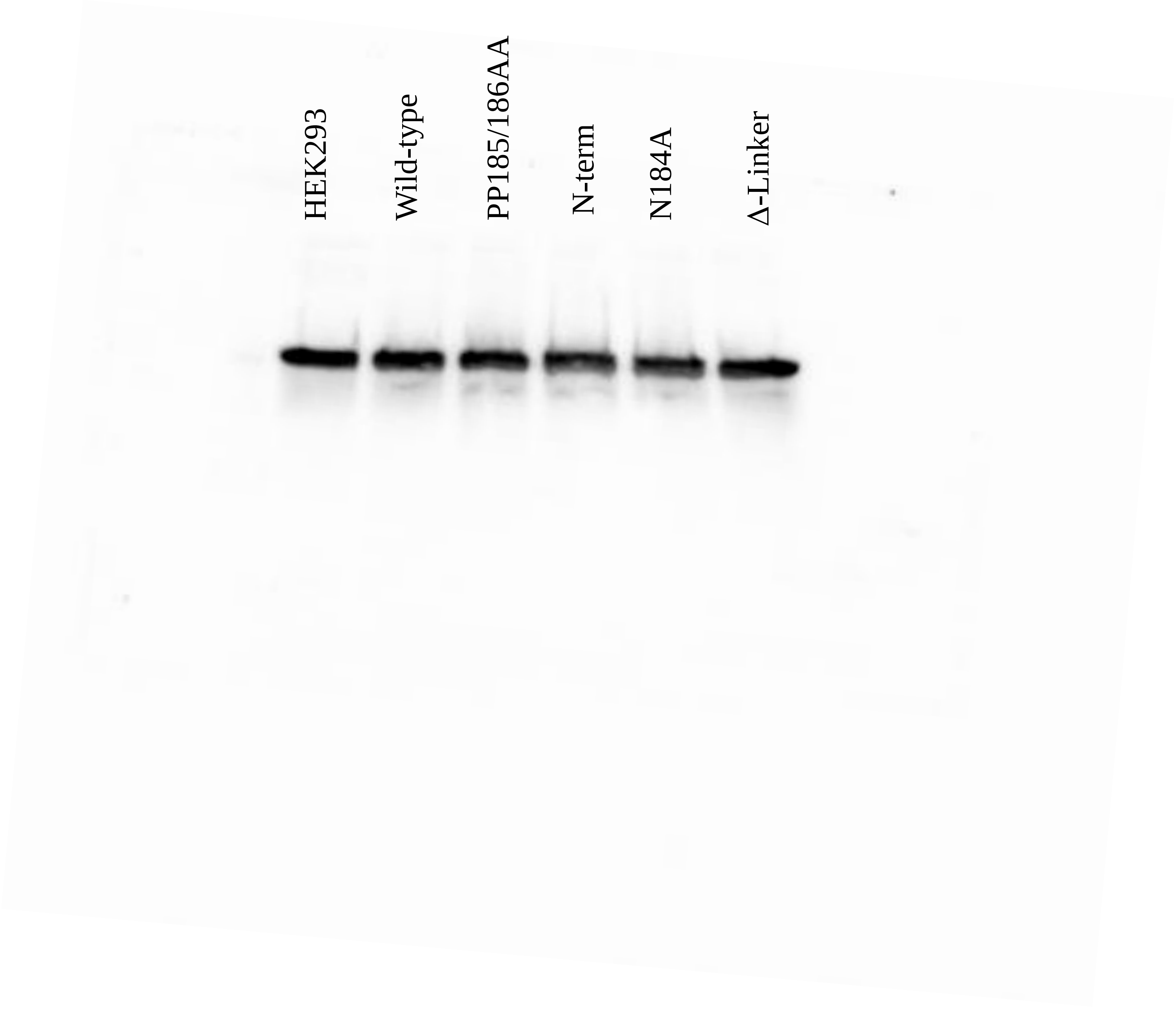

Figure 1B: MAT I/IIA

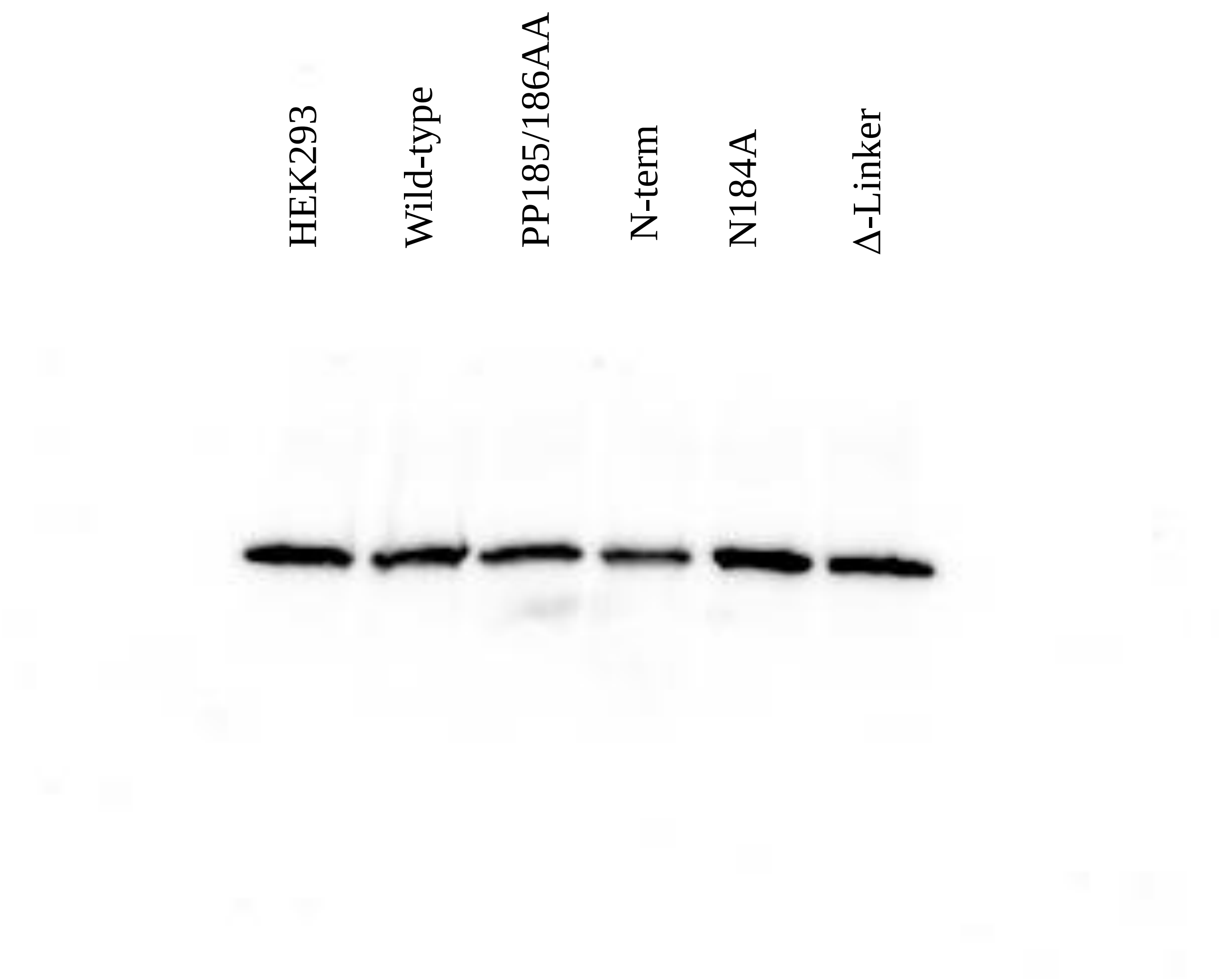

Figure 1C: METTL16

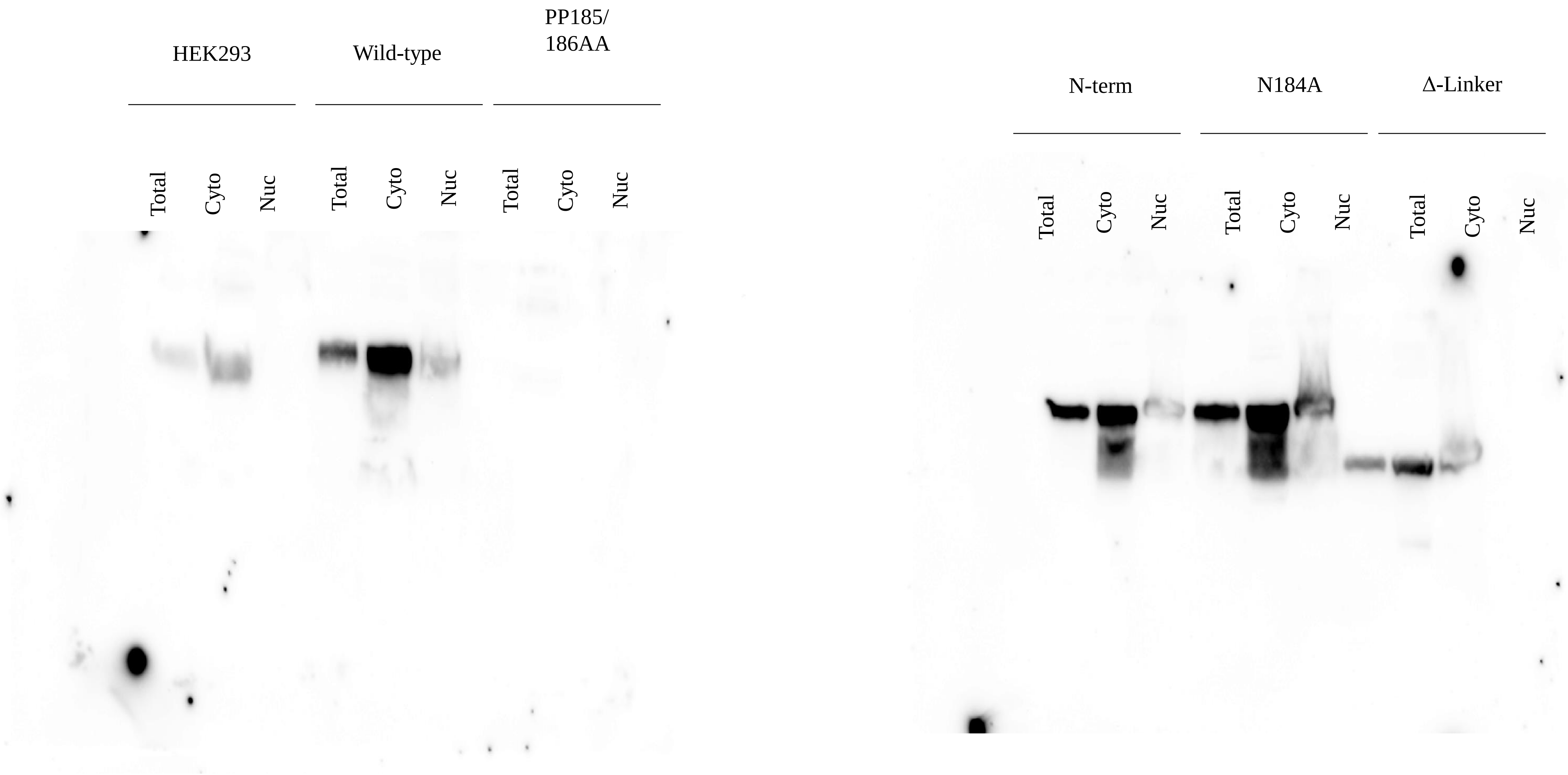

Figure 1C: Lamin B

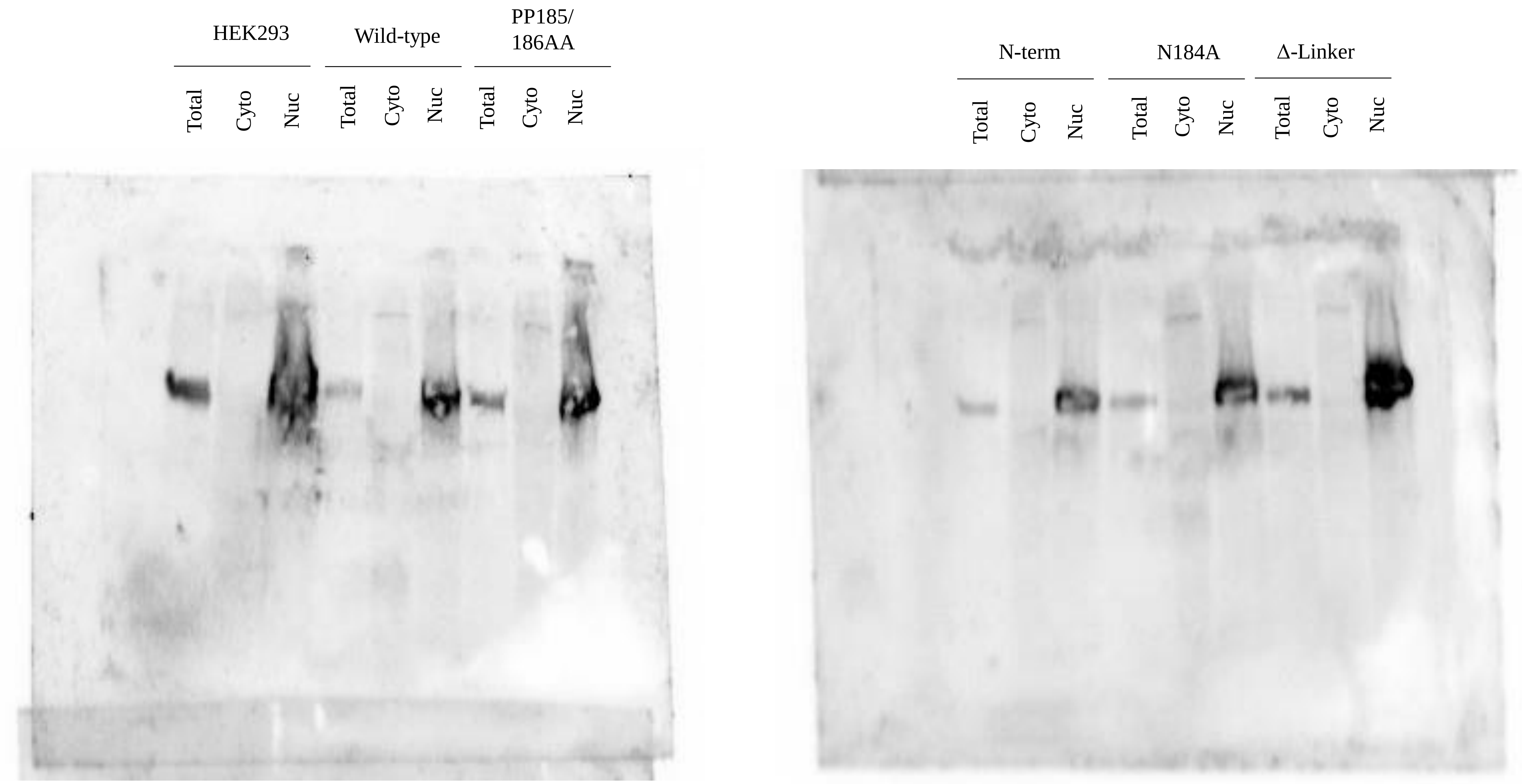

Figure 1C: LDH

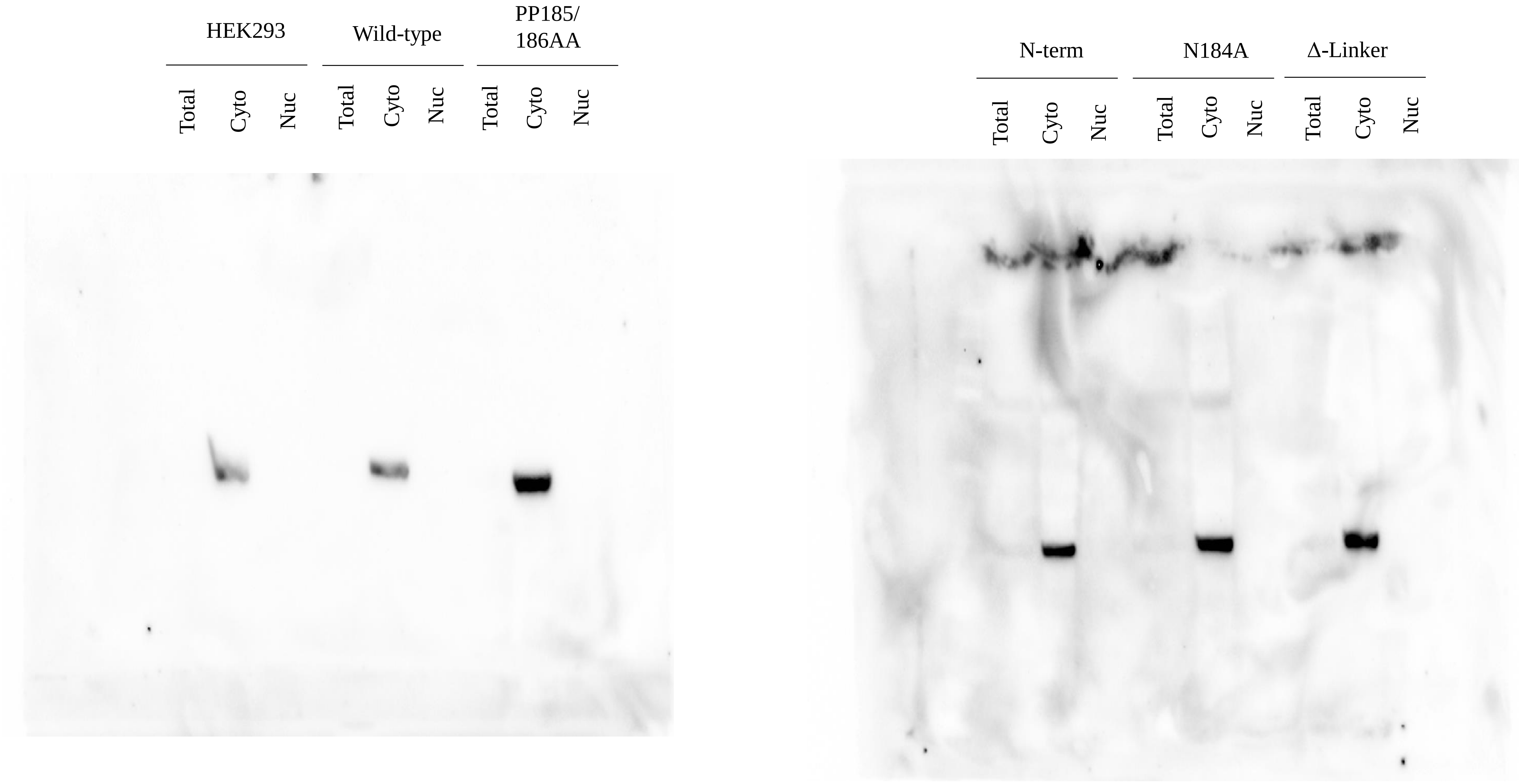

Figure 1D: HEK293

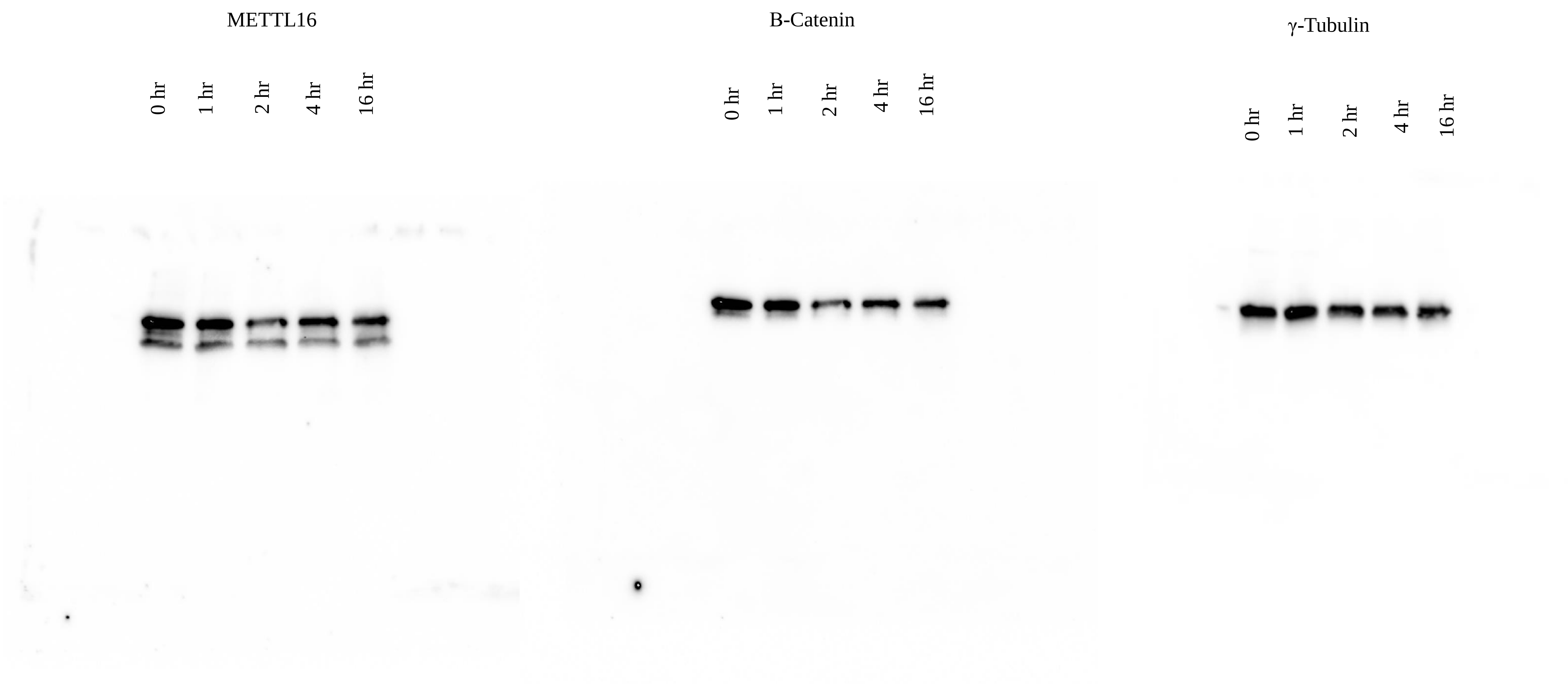

Figure 1D: Wild-type

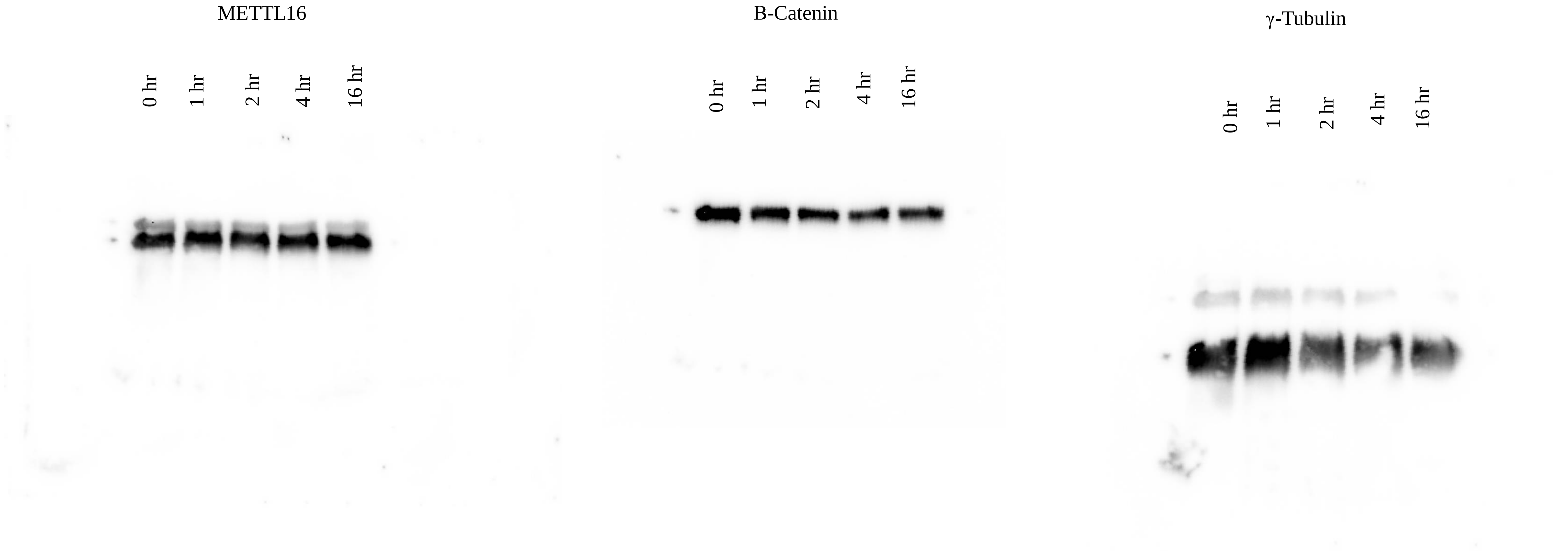

Figure 1D: PP185/186AA

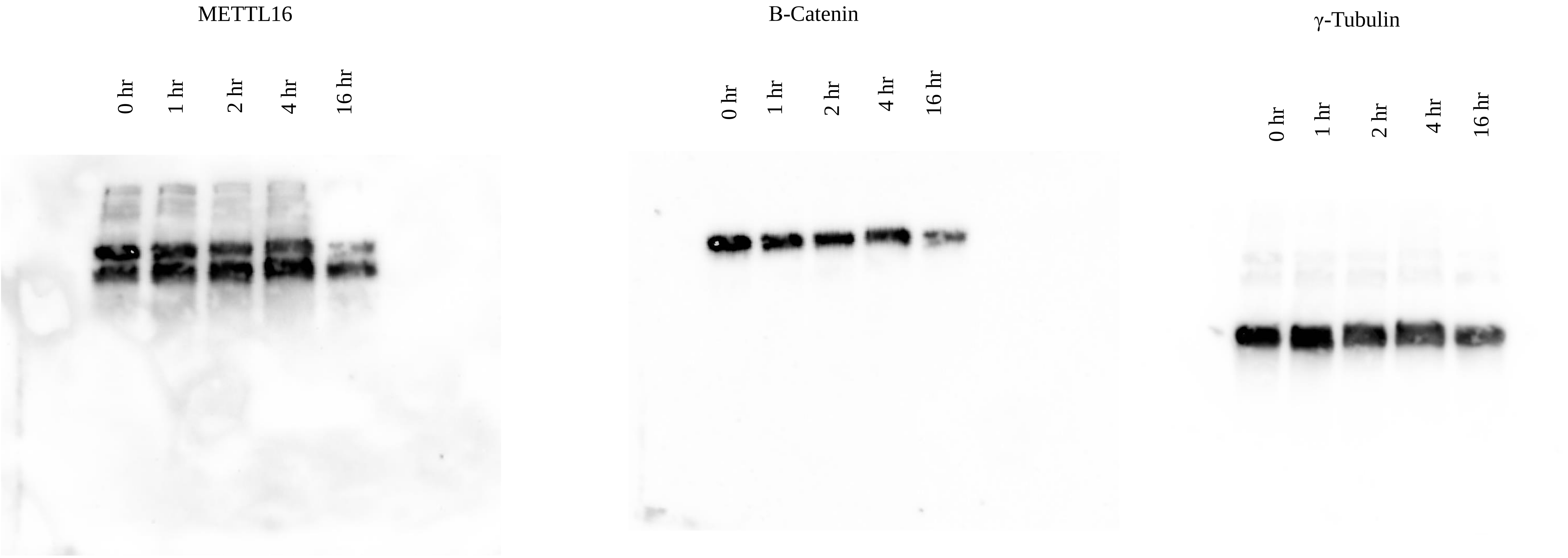

Figure 1D: N-term

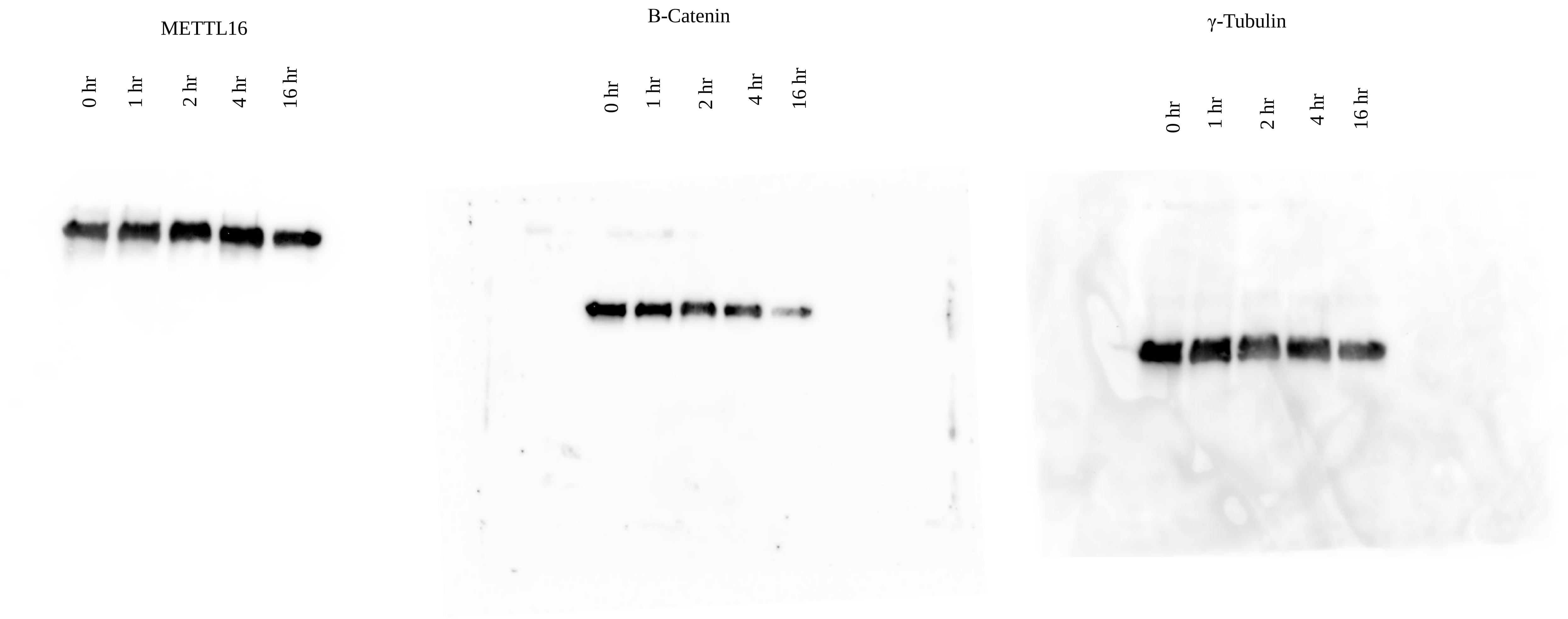

Figure 1D: N184A

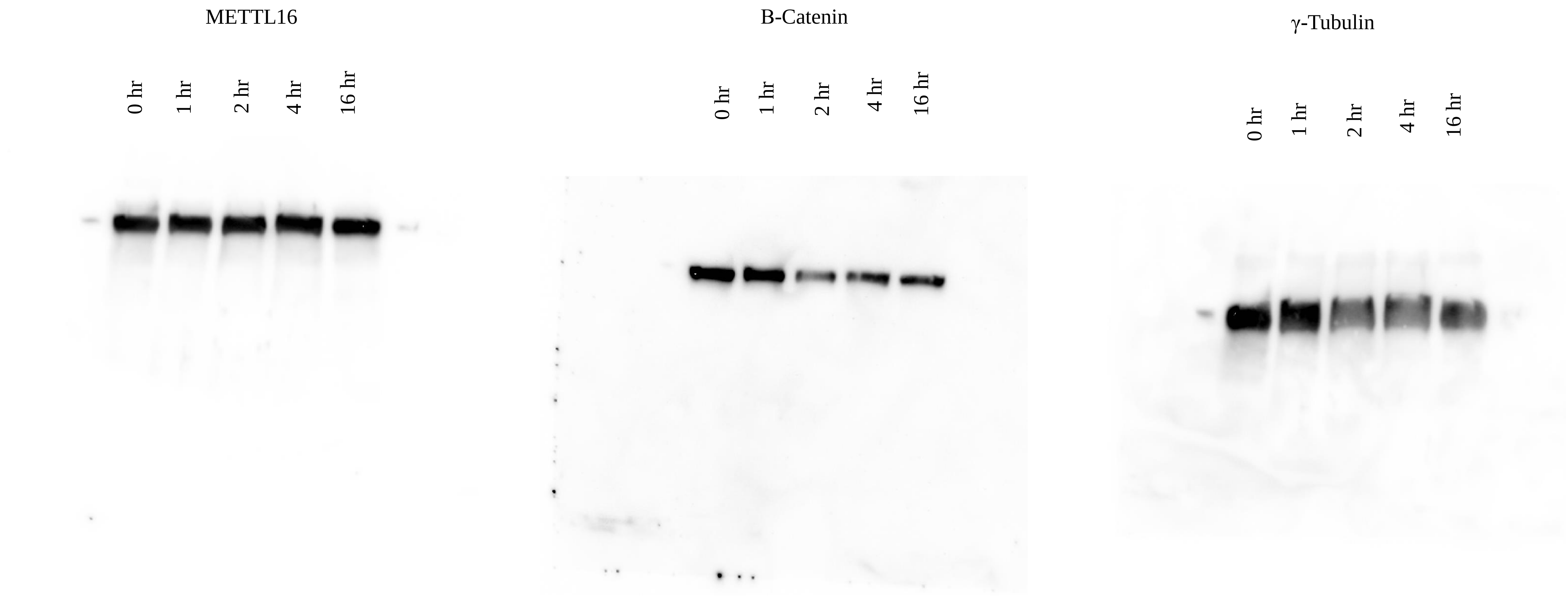

Figure 1D: Δ-Linker

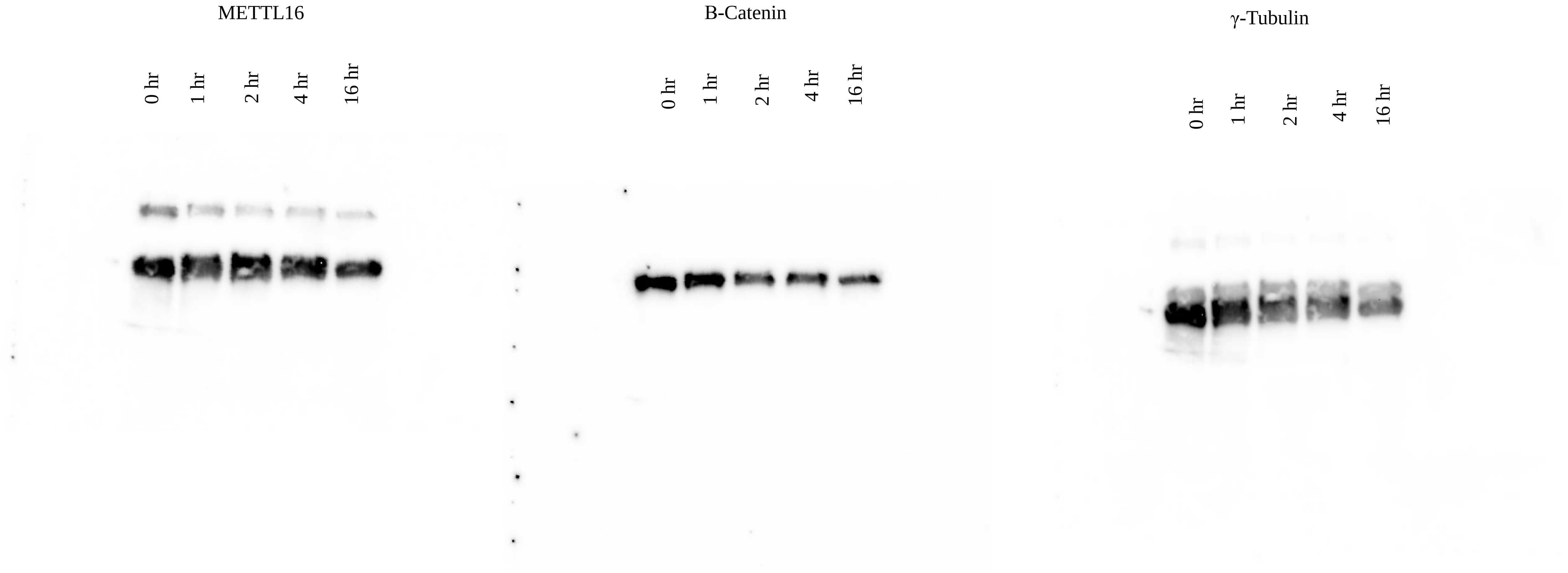

Supplement: Supplementary file 1 [file cimb-45-00346-s001.zip › Supplemental Figure 4_Original Western Blots.pdf]
