# Supplementary material for: RNA Methyltransferase METTL16’s Protein Domains Have Differential Functional Effects on Cell Processes
Source: Curr Issues Mol Biol. 2023 Jun 29;45(7):5460–80. doi: 10.3390/cimb45070346 (PMC10378215; doi:10.3390/cimb45070346)
Supplement: Supplementary file 1 [file cimb-45-00346-s001.zip › Supplemental Tables 1-4.pdf]

Supplemental Table 1: Antibodies used in this study

**Antibody**	**Vendor**	**Catalog #**	**Dilution**
Ɣ-Tubulin	Thermo Fisher	MA1-850	1:1000 (WB)
Lactate Dehydrogenase	Santa Cruz Biotechnology	SC-133123	1:250 (WB)
Lamin B	Santa Cruz Biotechnology	SC-6216	1:200 (WB)
MAT Iα/IIα	Santa Cruz Biotechnology	SC-166452	1:500 (WB)
METTL16	Bethyl Laboratories	A304-192A	1:2000 (WB)

Supplemental Table 2: PCR primers used to produce mutations in METTL16 sequence-containing plasmid

METTL16 Mutant	Site-Directed Mutagenesis Forward Sequence	Site-Directed Mutagenesis Reverse Sequence
CRISPR-Resistant	caacctcaacGGAAGAGTGAGCCTTAATTTTAAAG	atttgcacgtgCTGCTTAAAATCTGGATATTTG
N-terminal RNA binding	aatgcatacgcggacgcaCCTCCTGACTTTGCATATCTG	tgctgcatgcattgatgcACTCAGAGCCATGGCGAT
PP185/186AA Methylation	CATGTGCAACgcagccTTTTTTGCCAATC	CAAAAGTCATAGATTATCTCAGATTC
N184A Methylation	TTGCATGTGCcgcCCTCCCTTTTTTG	AAGTCATAGATTATCTCAGATTCTTC
Δ-Linker	GGAGTGGCCGGACAGTAC	CTTTTTCTCTTCCAAGGCCTGAATG

Supplemental Table 3: Plasmids and guide RNA sequences used in this study

**Plasmid**	**gRNA sequence**	**Catalog #**
Scrambled CRISPR	GGCTTCGCGCCGTAGTCTTA	CS-CCPCTR01-CG12-01-10
METTL16 CRISPR	CATGTTCAGATAAATCTGAA	CS-HCP218894-CG12-3-10-A
METTL16 ORF	-	RC208648

Supplemental Table 4: Real-time PCR primers used in this study

Gene	Forward (5'-3')	Reverse (5'-3')
ATXN10	GATACGATTGGTGTTGCTGTTG	GTCTATTGTTTGGGTGCATGC
CCND1	CATCTACACCGACAACTCCATC	CGGATGATCTGTTTGTTCTCCT
CDKN3	CCCAAACCTTCTGGATCTCTAC	TATGGAGGACTTGGGAGATCT
GPR50	GAATACCCACCGGCTCTAATC	TCTTCGTGGTCAGTCTCTCT
MALAT1	GAATTGCGTCATTTAAAGCCTAGTT	GTTTCATCCTACCACTCCCAATTAAT
MAT2A	CTGCTGTTGACTACCAGAAAGT	GCTACCAGCACGTTACAAGT
MYC	GCTGCTTAGACGCTGGATTT	GAGTCGTAGTCGAGGTCATAGTT
PNPLA4	CGTTGGTTGCTTCTGTTCTG	GACTAAGAAGTGGGATGGAGTC
RHO D	GCAGGGCAAGATGACTATGA	CAAGAAGGTACCCATCATCGT
RTRAF	ACAGGATGCAGTTGCTAAGG	TAGCTCTCTGAGCTCCTCTATG
U1	CCATGATCACGAAGGTGGTTT	ATGCAGTCGAGTTTCCCACAT
U6	CTCGCTTCGGCAGCACA	AACGCTTCACGAATTTGCGT
UBE2C	GCAGTTGCAGTCGTGTTCTC	TACAGCAGGAGCTGATGACC
